# Rapport a l'Academie Royale de Medécine sur la Peste et les Quarantaines, fait, au nom d'une Commission

**Published:** 1846-10-01

**Authors:** 


### MEDICO-CHIRURGICAL

### REVIEW.

OCTOBER, 1846.

I. Rapport a l'Academie Royale de Medécine sur la Peste et les Quarantaines, fait, au nom d'une Commission, par M. le Dr. *Prus*; accompagné de Pieces et Documents, et suivi de la Discussion dans le Sein de l'Academie. Ire et IIe Parties. 8vo. pp. 663. Paris, 1846. Bailliere.

II. Correspondence respecting the Quarantine Laws, since the Correspondence last presented to Parliament. Presented by Command to the House of Commons, in pursuance of their Address of May 19, 1846. Folio, pp. 48.

It must surely be quite unnecessary to say a single word in the way of soliciting our readers' patient and most attentive consideration of the facts and reasonings, which we are about to bring under their notice. The subject of the Quarantine Laws is one of public and very general interest. All persons are more or less immediately concerned in their operation and effects; for whatever interferes with the free and unrestrained intercourse of one nation with another, cannot fail to affect the common welfare. To the medical man the subject is, as a matter of course, doubly and trebly interesting; some of the most curious and important questions, connected with the natural history of epidemic diseases, are involved in its right adjustment. It is to medical doctrines and to medical opinions that we owe the present system of prohibitory restrictions, which so seriously interfere with the social comforts and commercial success of numerous countries; and therefore for this reason alone, if there was no other, it well becomes the members of our profession to be foremost in making a calm and candid examination of those doctrines and opinions from which such grave consequences have followed. Now, every one who has made himself acquainted with the subject, be he physician or merchant, traveller or statesman, has of late years, without exception, come to the decided conviction that it is high time for a thorough revision and a very material modification of the quarantine laws, such as they now exist, to take place. The absurdly foolish and most ridiculous principles which they embody, the vexatious and oppressive restrictions which they impose, the wretchedness and suffering which they almost necessarily give rise to, and the great increase of mortality which, we have reason to believe, they often occasion, are surely sufficient grounds for the scrutinizing investigation that is so generally demanded. For some years past, the British Government and that of France have been using their best exertions to effect a change, and have been trying to get the other Continental powers to co-operate with them in their good work. With this view, they have proposed that a Congress of Delegates from the different States of Europe should be held in Vienna, or elsewhere, for the purpose of agreeing upon some general and uniform system of Quarantine regulations to be adopted in the ports of the Mediterranean. Many difficulties, we regret to say, have been thrown in the way of this most equitable proposal by Prince Metternich, on the part of Austria, and by the representatives of some of the minor powers.* Will it be believed that, in several of the Italian States, the quarantine boards of health are actually independent of the government?—they possess a sort of patent vested right in the profligate exaction of their fees of office! Still, we must not be discouraged, nor diverted from the reform that is so loudly called for. Truth will assuredly prevail in the long run, despite the opposition of ignorant bigotry on the one hand, and of the basest mercenary intolerance on the other. We have only to keep the subject prominently and steadily before the public mind, by collecting accurate and well-authenticated facts from every quarter, and by not ceasing to expose the enormous fallacies and absurdities which prevail, and we may be confident that, ere long, the system which has so long existed to the serious detriment of commerce and to the disgrace of common sense, not to talk of science and humanity, will be made to undergo such changes as the present state of general enlightenment and sound knowledge requires.

It will be seen, from the following pages, that neither the French nor the English government has at all relaxed in their efforts for this most desirable end. Both have been accumulating materials for information, and collecting the opinions of competent authorities, in order that they may be ready to meet the objections or overcome the prejudices of their opponents.

In August 1844, the Royal Academy of Medicine in France appointed a Commission to examine all the varied questions connected with the Plague and with Quarantines. This Commission was composed of the following members—men, we may remark, of the highest professional and scientific attainments—MM. Adelon, Begin, Dubois (d'Amiens), Dupuy, Ferrus, Londe, Melier, Pariset, Poiseuille, Prus, and Royer-Collard. M. Ferrus was named the president, and M. Prus the secretary and reporter. The Commissioners were engaged in their deliberations for upwards of twelve months, and had every facility granted them by the French government, to render their enquiry as complete and as accurate as possible. At length, the report was drawn up and read at the sittings of the Academy, on the

---

* It is only doing justice to the Austrian government to state that it has already made some very useful practical reforms in its quarantine regulations. While France has been talking and planning, Austria, in imitation of the example set by this country, has been acting. Passengers from Alexandria can now reach Paris, *via* Trieste or Southampton, considerably sooner than by going to Marseilles, in consequence of their detention in the lazaretto there!

5th, 10th, 17th, and 24th of March and the 5th of May of the present year. It is certainly a very elaborate and instructive work, replete with most valuable facts and data, which cannot fail to be truly acceptable to every enquirer upon the great questions under consideration, whether he admits the soundness of the conclusions adopted by the majority of the Commission or not. It is for this reason that we have thought it right to bring before the attention of our readers, with as little delay as possible, a faithful summary of its contents, in order that they may be able to judge for themselves of the value of the original.

Although the Plague has so often ravaged the world and there has been no lack, as a matter of course, of books and memoirs published at different times upon the subject, it must be confessed that the number of instructive and really accurate narratives of well-observed *facts* is by no means very considerable. The epidemics, of which we have the most trustworthy histories, are the following:—that of Nimeguen in 1635, described by Diemerbrock; that of London in 1665, described by Sydenham and Hodges; that of Marseilles in 1720, by Chicoyneau, Verney, Deidier and Bertrand; that of Transylvania in 1755, by Chenot; that of Moscow in 1771, by Mertens, Orrœus, and Samoilowitz; and those of Egypt in 1798, 1799 and 1800, which have been so well described in the writings of Desgenettes, Larrey, Pugnet, and Louis Frank.

But, however valuable the records of the epidemics now mentioned may be, it must be admitted, we think, by all who have attentively studied the history of the plague, that it is only within the last ten or twelve years that we have anything like a positive and truly scientific acquaintance with the disease. Dr. Aubert-Roche was the first to display that brave and generous devotion to humanity and science, which has since been followed by so many of his professional brethren, when he brought himself in direct contact with his friend Dr. Fourcade, who died of the plague at Cairo on the 20th of February, 1835.*

Shortly afterwards, numerous plague patients were received into the hospital of Esbekiè, at Cairo. Clot-Bey, anxious to give the most complete authenticity to the observations which might be made of these cases, proposed to MM. Gaetani, Lacheze, and Bulard to join with him in forming a committee or board for the purpose of attending together upon all the patients in the successive stages of the disease, and of making *post-mortem* examinations. These four gentlemen carried through this task with the greatest zeal and devotedness. The infected were waited upon like other patients; they were freely touched whenever there was occasion to do anything for their relief, or for the investigation of their symptoms. The bodies of those who died were taken to the dissecting amphitheatre, and every organ was most attentively inspected. The results of each visit in common were carefully reported in a register, and each report was regularly signed by all four. This register (which was submitted to the perusal of the government Commission) is the chief basis of the works, which have been published by Clot-Bey† and Bulard.

---

* De la peste et du typhus d'Orient. Paris 1840, p. 90.

† This indefatigable person has sent no fewer than 50 memoirs, at different

Subsequently to these researches, the professors of the medical school at Abouzabel (about four leagues from Cairo) personally attended upon 140 plague patients, of whom 38 died. Professor Perron has communicated a report of the observations and *post-mortem* examinations then made, in a memoir which he addressed to the Academy.

Drs. Aubert-Roche and Rigaud, attached to the great hospital at Alexandria, displayed no less courage and disinterestedness in their enquiries. The latter gentleman died of the plague, leaving behind him an account of 68 dissections which he had made of fatal cases.* The former has published an account of his observations, collected either by himself or in conjunction with his lamented colleague.

The conduct of M. Lesseps, the French consul-general at Alexandria, has been the theme of universal admiration. By his own example, he powerfully contributed to dissipate the exaggerated apprehensions of visiting and even touching plague-patients. His conduct towards Dr. Rigaud, up to the last moment of his friend's life, was a memorable instance of noble generosity.

Since 1835, the medical men resident in Egypt have continued their efforts to render our knowledge of the plague more and more complete. In 1837, an epidemic broke out at Adana, in the corps of the Egyptian army that then occupied Syria. In 1841, Damietta, Cairo, and a number of the towns or villages in the Delta were visited by the pestilence. It is also to be remembered that not a year has passed since the great epidemic of 1835, without a greater or less number of sporadic cases occurring every now and then in different parts of Lower Egypt.

But the plague has been studied of recent years in other countries besides Egypt. To confine our notice to modern works only, we may mention Dr. Brayer's *Neuf années à Constantinople ;* Dr. Gosse's account of the plague in Greece during 1828 and 1829 ; and the reports of Dr. Morea on the plague of Noja in 1817, and of M. Hemso on that of Morocco in 1818.

M. de Segur du Peyron, although not a physician, has rendered great services to medicine by the publication of the three reports which he addressed, in the years 1834, 1839, and 1846, to the minister of commerce, and which contain a great mass of observations collected by him in the principal ports of the Mediterranean.

Lastly, the Academy has received a memoir on the plague and quarantine, published in 1845 by Dr. Moulon, physician of the lazaretto at Trieste ; and also a printed report on the transmission of the plague and the yellow-fever, that was drawn up by a committee of the medical society of Marseilles, and unanimously approved of and adopted in August 1845.

Besides the published works above enumerated, a number of very valu-

---

times, on the subject of the plague to the French Academy. Many of these memoirs, written by able men who have had ample opportunites of studying the disease, well deserve to be published.

* In the very valuable pamphlet on Oriental Plague and Quarantines, published by Dr. Bowring in 1838, it is stated that Dr. Rigaud, after having been, during the most fearful crisis of the pestilence (1835), constantly engaged in visiting and assisting the living or in dissecting the dead, at length fell a sacrifice "just as the plague was ceasing, when its violence appeared wholly exhausted, and the season of its disappearance was about to arrive."

able manuscript documents have been submitted to the examination of the Commissioners.

Among these, we may mention the original papers respecting all the cases of plague that have occurred in the lazaretto of Marseilles since 1720, along with a letter and memoir from Dr. Robert, one of the physicians of this lazaretto;—the register kept in Egypt and Syria, during the years 1828, 1829 and 1830, by the plague commission, of which M. Pariset was the president;—the report, addressed in 1842 to the minister of commerce, by Dr. Delaporte of his mission to Constantinople, Smyrna, and Alexandria, for the purpose of studying the plague in these places;—a statistical statement of 506 epidemics of the plague drawn up by Dr. Rossi of Cairo, who, like Dr. Delaporte, nearly fell a sacrifice to an attaek of the pestilence;—the statistic report of all the cases of plague observed in the lazaretto of Alexandria since 1835, by Dr. Grassi, who has been physician of that establishment since 1831;—a memoir on the plague in Persia by Dr. Lacheze;—one on the plague in Algeria, from the year 1552 down to 1819, by M. Berbrugger, corresponding member of the Institute, and conservator of the library and museum of Algiers;—a memoir on the contagiousness of the plague by MM. Pezzoni, Leval, and Marchand, members of the council of health of the Ottoman empire, dated June 1842;—and lastly, a memoir on the antiquity and endemicity of the plague in the East, and especially in Egypt, by Dr. Daremberg, the learned librarian of the French Academy.

In addition to these numerous sources of information, the Minister of Foreign Affairs granted to M. Prus the privilege of consulting the dispatches of the French ambassadors and consuls in the Levant on all topics connected with his enquiries. The dispatches of M. Lesseps, (to whom we have already alluded,) during the frightful epidemic of 1835 in Egypt, were found to be especially valuable. The Minister of Marine also put all the official documents under his control at the free disposal of the Commissioners.

With the view of rendering their enquiry as complete and comprehensive as possible, the Commissioners invited to their meetings the attendance of medical men and others, who might feel inclined to give any verbal communication. In this way, they received much valuable and interesting matter.

The Report is divided into four parts or sections.

In the *first*, the following points are examined and determined:—the countries where the plague has been observed to become spontaneously developed;—the causes of spontaneous plague;—the disappearance of the plague, whenever these causes have ceased to exist;—the countries where the persistance of these causes renders the plague endemic, or at least makes the return of the spontaneous disease to be apprehended—and, lastly, the measures that are really and truly prophylactic against spontaneous plague.

In the *second* part, the three following questions are answered:—1. Has the plague always exhibited the characteristic features of epidemic diseases, whenever it has raged in Africa, Asia, and Europe? 2. What are the distinctive characters between epidemic and sporadic plague? 3. Does the plague spread after the manner of epidemic diseases; *i. e.* by the migration of certain atmospheric influences, and independently of the agency of those persons who are infected by it?

In the *third* part, the important question as to the transmissibility of the plague from one individual to another is examined. Is the disease transmissible by inoculation? Is it transmissible away from, as well as in, epidemic *foci* by immediate contact with the sick? by the contact of clothes, furniture, or merchandise? or by miasms exhaled from the bodies of the sick, and diffused through the atmosphere? This part closes with an examination of the following three questions. 1. Can persons affected with sporadic plague occasion *foci* of infection sufficiently active for the transmission of the disease? 2. Is the plague more or less readily transmissible, in proportion to the intensity of the epidemic; according as the disease is in its first, its second, or its third period; and, lastly, according to the organic susceptibilities of those who are exposed to the action of the pestilential miasm? 3. If the plague be transmissible away from epidemic *foci*, are there any grounds to apprehend that the importation of a few cases into France might occasion a pestilential epidemic?

In the *fourth* and last part, the question as to the ordinary or exceptional duration of the incubation of the plague is discussed. The general conclusions of the Report, and the application of these conclusions to the important subject of Quarantine are appended to this part.

### First Part.

Chap. I.—*What is the country, or what are the countries, where the Plague has been observed to arise spontaneously?*

In attempting to trace back the history of the plague, with the view of throwing some light upon this question, it would be little profitable to carry our researches beyond the sixth century of the Christian æra, as there is too good reason to believe that the terms λοιμος and *pestis* were previously used in a generic sense, to denote all epidemic diseases which caused great mortality. The "boils breaking forth with blains upon man and beast," recorded by Moses, need scarcely be alluded to. The famous plague of Athens, so graphically described by Thucydides, is supposed by the best authorities to have been a malignant form of typhus, complicated with a peculiar eruption and with gangrenous eschars. The Greek historian says that it was believed in his day that the pestilence had been imported from Egypt into the Piræus.

Whether we are to admit the genuineness of the passage from Rufus of Ephesus, a celebrated physician in the time of Trajan—discovered by Cardinal Angelo Mai, at Rome, in 1831, in the writings of Oribasius, who lived in the time of the Emperor Julian—is doubted by some learned enquirers; by M. Pariset, the accomplished secretary of the Academy, among the number. The passage indeed contains a remarkably accurate description of the characteristic symptoms of the plague,* and the writer refers to

* The following is one of several paragraphs that might be quoted:—
"A pestilential carbuncle is that which is accompanied with a severe inflammation, with acute pain, and delirium. In many of those who are affected with it, there occur also hard and painful bubos, and the patients soon die of these carbuncles. This is the case more especially with those who live in the neighbourhood of marshes."

epidemics of the disease in Egypt, Syria, and Libya, mentioned by Dioscorides, Posidonius, and Dionysius, who (are supposed to have) lived two or three centuries before the Christian æra. It may be worthy of notice here that the writings of Cicero, Strabo, and Pliny afford evidence that Egypt was regarded, in their time, as a country that was fertile in the plague. There are allusions too in the works of Galen and Aretæus, not to mention Hippocrates, that would seem to indicate their acquaintance with malignant fevers accompanied with bubos and carbuncles.

But, without dwelling longer on the uncertain history of the plague, we shall at once come down to the year 542 of the Christian æra, when that terrible epidemic, the description of which by Procopius and Evagrius can leave no doubt as to the true nature of the disease, ravaged the city of Constantinople. From this period, the appellation has been very generally restricted to that form of fever that is accompanied with bubos, carbuncles, and petechiæ. If we are to believe that from the 6th to the 16th century the term "plague" has been properly applied, we are surely justified in assuming that, from the beginning of the 16th century—that is to say, subsequently to the establishment of lazarettos in Europe—this word has only been employed in its right acceptation.

In the 16th century, there was (as far as we know) but one epidemic of plague in Egypt, and we find no mention of any in Turkey in Asia, or in Syria; whereas, in the course of this century, there were no fewer than fourteen invasions of the pestilence in France, twelve in Germany, eleven in Italy, nine in Dalmatia, six in Turkey (in Europe), five in England, five in Spain, two in Portugal, two in Poland, two in Belgium, and one in Switzerland.

In the 17th century, we have the account of but two invasions in Egypt, and of not one in Turkey (in Asia) or in Syria; whereas there were nineteen in Germany, eleven in Italy, eleven in France, six in England, five in Russia, four in Turkey (in Europe), three in Spain, two in Holland, two in Switzerland, two in Denmark, one in Sweden and one in Poland.

It seems, therefore, impossible that any one, who will take the trouble of comparing these figures (always supposing that they can be depended upon—*Rev.*) should not be struck with this remarkable circumstance; to wit, that the plague has repeatedly and most destructively made its appearance in many points or localities in the world, more especially in Europe, at various epochs when either it did not exist, or was only very rare, in Egypt. If such has been the case, we are surely bound to admit that the disease has often arisen spontaneously in other countries, besides Egypt, Turkey, and Syria. This is the view which M. Littré has taken; for he observes (article *Peste* in the Dictionaire de Medecine) that "the plague was very frequent in Europe during the 16th and 17th centuries. Italy, France, England, Holland and Germany were attacked by this pestilence; and Paris and London witnessed it spring up in the midst of them just as Cairo and Constantinople now do."

In the 18th century, epidemic plague occurred nineteen times in Egypt, seven times in Turkey in Europe, four times in Dalmatia, four times in Germany, thrice in Russia, thrice in Spain, twice in Poland, twice in Greece, once in Italy, once in Sweden, and once in France, viz. when Provence and Marseilles suffered so severely in the years 1720 and 1721.

In the course of the present century, the epidemic plague has broken out eight times in Egypt, six times in Turkey in Europe, thrice in Greece, twice in Syria, twice in Italy, twice in Russia, once in Turkey in Asia, once in Germany, once in Dalmatia, and once in Morocco.

The statistical data which we have given, more especially those which have reference to the 16th and 17th centuries, appear to prove most incontrovertibly that the plague has arisen spontaneously, at certain periods, in very many of the countries of Europe and Asia. Our convictions upon this point will be much strengthened, if they are found to be supported by facts observed in our own day.

Dr. Lacheze, in the account of his recent travels through Persia, informs us that the plague has been repeatedly observed to arise spontaneously in several of the cities of Asia Minor, and particularly at Erzeroum, situated near the northern source of the Euphrates and about five days' journey from Trebisond; a statement which has been amply confirmed by the report of the Turkish council of health, that was established in the year 1838. The same remark may be made respecting Aleppo.

There are numerous facts also that seem to prove that the plague is apt to appear spontaneously upon the banks of the Danube, as it does on those of the Nile and the Euphrates.

The Russian army in 1828, while engaged in war with the Turks in Moldavia, Wallachia and Bulgaria, was attacked with a very malignant fever that was accompanied with bubos in the groins and axillæ. Dr. Witt, principal physician of this army, while he has acknowledged that the fever resembled in every respect the true plague, gave it, however, as his opinion that it must be distinguished from this disease, because it arose on the banks of the Danube, independently of any importation from abroad! Dr. Schlegel, who had been sent by the Russian government, before the arrival of Dr. Witt in Wallachia, to determine the nature of the epidemic admitted that, although it shewed great affinity to the plague, it differed from the latter in being attributable in that country to putrid emanations containing mephitic gas! On the other hand, Professor Seidlitz of Petersburg did not hesitate to regard the fever as genuine oriental plague.* If it was really so—and surely there is no good reason to think otherwise—we have the authority of both Dr. Witt and Dr. Schlegel that the disease was truly endemic and of spontaneous origin in the localities where it prevailed.

Although the plague may therefore arise spontaneously in a number of different localities, it is no doubt true that, in recent times, Egypt, Syria, and Constantinople—more especially the first—have been the principal *foci* of the disease. It is right here to mention that, since the year 1839, it appears that there has been no case of plague observed in Constantinople. The board of health of that city attributes this exemption altogether to the quarantine measures, that have been adopted of late years. May

---

* This gentleman has shewn that, whenever in times past the Russians have carried on war against the Turks on the banks of the Danube and on the coast of the Black Sea, their armies have almost invariably suffered from the plague. In consequence of this fact, and other considerations to be afterwards mentioned, he does not hesitate to affirm that, in these cases, the plague is to be viewed as only the worst form of the endemic fever of the country.

such be the truth! but let it not be forgotten that, before the terrible epidemic of 1812, not one case of the disease had occurred in that immense city for eight years;—a fact that is proved by the registers of the French embassy at the Sublime Porte.

Syria also appears, according to the testimony of Mr. Lander the English Consul at the Dardanelles, and of M. Beclard the French Consul at Smyrna, to have been completely exempt from the plague since the same period (1839). Dr. Lasperanza, attached to the Constantinople board of health, informs us that, in consequence of various sanitary improvements that have of late years been introduced, the disease has ceased to be endemic in Jaffa, as well as in other parts of Syria. In the present day, it is almost exclusively from Egypt that the importation of the plague may be apprehended.

The general *conclusion* from all that has now been stated is that—

"The plague has been observed to arise spontaneously, not only in Egypt, Syria, and Turkey, but also in many other countries of Africa, Asia and Europe."

CHAP. II.—*In countries where the spontaneous plague has been observed, can the development of the disease be reasonably attributed to any determinate hygienic conditions?*

To solve this question, the Commissioners examined with great care the history of the various localities in which the plague has arisen spontaneously, within the last fifty years. And first with respect to Egypt. Now the most competent observers assure us that there is nothing in the mere climate of this rich, and, in many respects, highly favoured country that will account for the generation of the pestilence; indeed, travellers have written in the most glowing terms of its beauty and salubrity. The year in Egypt may be divided into three periods or seasons. The *first* commences in August and ends with October; it is the period of the inundation of the Nile. The *second* comprises the next six months, from November to April; it is the season of the winter harvests, the ground being covered with trefoil, wheat, barley, flax, &c. The *third* begins in May, and terminates in August or September; it is the time for the cultivation of cotton, indigo, and rice. As we have already said, the natural climate of Egypt is on the whole a very salubrious one. Its drawbacks are but few; the chief being the coolness and humidity of the nights, the frequent and rapid variations of temperature in the day, the rains and fogs of the Delta during the winter months, the great heat and excessive dust in summer, and, lastly, the singular effects of the South wind, the *Kamsin*, upon the living body. Whence then comes the pestiferous atmosphere of some parts of this land? The answer is ready; man himself has given it birth; the inhabitant of the Delta, says M. Hamont* who long resided in Egypt, has prepared the causes of his own destruction. The destitution, filth, and misery of the poor inhabitants are extreme. Their wretched hovels are so horribly disgusting as almost to defy description; they are not only surrounded by, but are actually receptacles of, heaps of ordure and putrid matters. Not

---

* *Destruction de la peste et des quarantaines.* (*Bulletin de l'Academie Royale de Medecine,*—Paris, 1844, t. x. p. 40.)

unfrequently the dead are buried immediately under the mud floors of these dwellings of the living; and many of the graves in the cemeteries (which are always within the villages), being left open, are continually exhaling a stench that is utterly intolerable to any stranger. Then, again, the food of the Fellah is always of the worst description, and often too of the most scanty supply. Rotten cheese, decayed vegetables, semi-putrid flesh or fish; such are the articles that he lives upon. The very water that he drinks is filthy and impure. And then think of his mental and moral condition; the brutish degradation of all his faculties and affections, his hopeless servitude, his blank unmitigated wretchedness.

The hygienic state of the cities and larger towns in Egypt is not much better than that of the villages. Cairo, with its 200,000 inhabitants, is a very hot-bed of the most disgusting and pestiferous impurities. From the canal, which traverses it, there is constantly steaming forth a cloud of intolerable offensiveness; and yet this is the supply of water for the use of its people! There are no fewer than 35 cemeteries, of which 25 are within its walls. In the Copt quarter of the town, the dead are buried under the floors of the houses; and nothing but a few boards separate the living from the putrid bodies of the deceased. From 80 to 90 corpses have been known to be huddled together in these horrible *sub-domal* receptacles. Can we therefore wonder that Cairo should be a generating focus of pestilential disease?

That the circumstances now mentioned must tend to promote the development, and aggravate the intensity, of the plague will be disputed by none; but then the question comes to be, are they sufficient to produce or originate it? This thing is certain, that the disease has never been known to appear spontaneously in Egypt, except in places and seasons when these most pernicious agencies were at work.

The plague does not arise in Upper Egypt, Nubia, and Abyssinia; nor does it ever extend above the first cataract of the Nile. The good quality of the soil, the ready efflux of the waters, the small number of the inhabitants, and the strong currents and agitations of the atmosphere appear entirely to counteract the morbid influence of the mode of life followed by the inhabitants.*

We are informed by Gaetani Bey†—first physician to Mehemet Ali, and who has resided in Egypt for the last 25 years—that the plague never extends beyond Assuan, in consequence of the difference in the situation, heat, dryness, and nature of the soil; whereas it readily finds its way into the localities where there is much stagnant water. It is for this reason that Bagdad and Bussorah are in the present day subject to invasions of the pestilence, from which they were formerly exempt when effective police regulations were in force in these towns.

The seasons exert a no less marked influence on the development of the plague. The dry heat of what is called in Egypt the second summer, the prevalence of the northerly wind that usually sets in about the summer solstice, and the first dews that commence about this time, change alike the condition of the atmosphere and the organic aptitudes or susceptibilities: the pestilence ceases.

---

* Pariset, *Causes de la peste.*—Paris, 1837.

† *Sulla peste che afflisse l'Egitto, l'anno* 1835. *Napoli* 1841.

What has been now said respecting the artificial insalubrity of Egypt, arising from man's own negligence and vice, is nearly quite as applicable to Constantinople as it is to Cairo. The filth of some of its environs is altogether intolerable and disgusting. It is usually in the month of July, when the north or tramontane wind ceases and is succeeded by a southerly sirocco, that the pestilence makes its first appearance. As a matter of course, the putrefaction of all organic matters goes on much more actively at that season, in consequence of the high heat on the one hand, and the moist relaxing influence of the wind on the other. The localities, that are first attacked, are those which are chiefly occupied by the poor Greeks and Jews: hence the village of San Dimitri is usually the place where the earliest cases are observed.

We need scarcely say that, if Constantinople be bad, Erzeroum is much worse, in everything that respects hygienic salubrity. Fortunately the frequent severity of the winter season there, as well as the high winds that prevail in Armenia, tend much to attenuate the existing causes of the plague.

If from the Euphrates we pass to the Danube, we shall find the same causes of endemic insalubrity prevailing in those localities, where the pestilence has been known to arise. The poorer classes in Moldavia and Wallachia live in the greatest misery and filth. After the heats of summer, almost all the prevailing diseases assume a character of marked gravity. Malignant intermittent fevers are always more or less prevalent in autumn; these generally precede the appearance of the plague, which in these countries is usually only sporadic. Professor Seidlitz has endeavoured, as we have already seen, to establish the intimate connection between these two forms of febrile disease.

Dr. Mirolanof, who treated the plague at Achial in 1828, says that "the soldiers and officers, who had the intermittent fever, were affected with bubos and carbuncles. In the month of September the plague shewed itself especially in those who were convalescent from agues, and assumed the form of a tertian fever. The bubos appeared after the first or second paroxysm."

Dr. Rinx, who was at Adrianople during the whole course of the epidemic, remarks of the third degree of the epidemic that "the least severe degree of the plague so much resembled an intermittent fever that it was scarcely possible to distinguish the one from the other, before the appearance of the bubos."

From all these various facts, it is abundantly obvious that the hygienic condition of the four distinct localities, in which the plague has of recent years broke out spontaneously, is very nearly the same. It is a circumstance, too, of no trifling import that, wherever the producing causes of the disease are most abundant and concentrated, there it is always most severe and most readily propagable. The form most dreaded is that which appears in Egypt; next comes that of Constantinople, and after this that of Erzeroum; while that of the Danube, which has hitherto been generally regarded as of Constantinople growth, has not yet been sufficiently studied to enable us to decide respecting its relative severity.

Is it not also a remarkable fact that the four geographical points or localities, now mentioned, are all subject to malignant intermittent and other fevers? Are we to believe, with MM. Begin and Boudin, that the plague belongs to the family of marsh fevers? There are many circumstances certainly which seem to militate in favour of this opinion. Without dwelling on the geographical condition of Syria and other plague countries in the present day, we well know how prevalent intermittent fevers were in London during the 17th century, when that city was occasionally visited by the oriental pestilence. The readers of Sydenham are well acquainted with this fact.

The outbreak of the plague has not unfrequently followed upon wars, famines, and other wasting calamities; and, on the other hand, its ravages have invariably been observed to become less frequent and less desolating in proportion as the condition of the inhabitants of the affected countries, in point of civilization and comfort, has improved. The researches of MM. Papon* and Aubert-Roche† have satisfactorily proved the truth of this.

The general *conclusion* to which we arrive is that,

"In all countries where the spontaneous plague has been observed, its development may be reasonably attributed to certain determinate conditions acting upon a large portion of the inhabitants. The principal of these conditions are, residence upon marshy alluvial soils near the Mediterranean or near certain rivers, as the Nile, Euphrates, and Danube; the dwellings being low, crowded, and badly ventilated; a warm moist atmosphere; the action of putrescent animal and vegetable matters, unwholesome and insufficient food; and great physical and moral wretchedness."

Chap. III.—*If the preceding statements be correct, the plague must be endemic in Lower Egypt, where all the conditions of insalubrity which we have pointed out are constantly present: Is such the case?*

All the most accurate and enlightened observers agree in answering this question in the affirmative. Not a year passes without the plague shewing itself at Alexandria in a *sporadic* form; generally between the months of November and the following June. This fact cannot be disputed; it is incontrovertibly proved by the reports of the Council of health that was established in that city 12 years ago. The same thing holds true with respect to Cairo, and other places in Lower Egypt; the testimony of Gaetani-Bey is unqualified upon this point. The *epidemic* plague, that which has so fearfully mowed down the Egyptian population, is happily more rare; although incomparably more frequent than in any other country of the world.‡ The number of epidemic invasions of the pestilence in Egypt from the year 1695 to 1834, have been (according to one statement drawn up by an Arab cheik) 19 in all. The mortality caused by some of the invasions has been truly frightful. If we take account of those only

---

* *De la peste et des epoques memorables de ce fleau.* Paris, an. viii. 2 vol. 8vo.

† *De la prophylaxie de la peste,* Paris, 1843.

‡ According to the calculations of M. Hamont, the population of Egypt, which was once, it is believed, upwards of ten millions, and was fully three millions at the commencement of the present century, does not now exceed a million and a half.

which have been very destructive of life, we find that Egypt has been visited with the scourge about once in every ten years.

*Conclusion.*—" All the producing causes of the plague being found united in Lower Egypt, the disease is endemic in that country, where it is seen every year in the sporadic, and about every tenth year in the epidemic, form."

The object of the next Chapter, the IVth, is to shew that Egypt was exempt from pestilential epidemics in ancient times, and until about the commencement of the seventh century of the Christian æra. Certain it is, that we have no very authentic account of any wide-spread and destructive invasion of the plague at an earlier period. The cases, alluded to by Rufus, appear to have been only *sporadic;* at all events, this writer makes no distinct mention of any epidemic pestilence having ever prevailed in Egypt, as he does of one that ravaged Libya upwards of 300 years before the birth of Christ.

That Egypt was once a remarkably healthy country is expressly attested by Herodotus. The land was rich and very populous, abounding in all the necessaries of life, and the inhabitants were prosperous, enlightened, and happy. The custom of embalming the dead, not human beings only but animals of all sorts, may have had a salutary influence, by withdrawing so much corruptible matter from putrefaction and decay.* This " salutary practice" (of embalming) was abolished in A.D. 356.† Subsequently to this period, the ignorance and fanaticism of the Mussulmen have brought on that frightful state of moral degradation and physical wretchedness of which we have spoken in a preceding chapter. Is this lamentable state of things always to last, to the disgrace of the country and the injury of the world? There cannot be a reasonable doubt but that, if proper sanitary regulations could be established and duly executed in Egypt, the pestilence might be extirpated, and Egypt rendered as healthy as it was in days of yore. Mehemet Ali is well aware of the truth of this. His convictions on this point are so positive, and already he has acted so well in the right course, that Gaetani-Bey—who accompanies him twice a year in his tours of inspection across the Delta, from Alexandria to Cairo—has not hesitated to declare that, if the Viceroy was not thwarted in the execution of his plans, this great and desirable end might be accomplished.‡

* There seem to be some inconsistencies between a few of the statements in this chapter and those that have been already made, respecting the existence of the plague in Egypt in ancient times. We may remark also that the testimony of Herodotus, respecting the salubrity of ancient Egypt, is said to be at variance with that of other authors more worthy of credit. M. Daremberg informs us that Hæser (*Recherches historico-pathologiques sur les maladies epidemiques*) has collected together a number of texts to prove the unhealthiness of Egypt in ancient times. Compare also Lorinser, *die Pest* im *Orient.*

† M. Prus suggests, among other hygienic reforms necessary in modern Egypt, the re-establishment of the practice of embalming (!) or of some other equivalent method of counteracting the evils of animal putrefaction in that country.

‡ Sir W. Pym, in a letter addressed by him in Jan. 1845 to the Board of Trade, acquaints us that Mehemet Ali, on being informed that there was a very short quarantine in England against Egypt, replied: "There ought to be no quarantine, it is our own fault. *We must get rid of the plague !*"

The question proposed in Chapter V. is to the effect whether the present condition of Syria, of Turkey in Europe and Asia, and of the Barbary States has become so much changed or ameliorated, since the time when pestilential epidemics have broken out in them, as to justify any rational expectation that such invasions may not recur. The answer, as might be anticipated, is decidedly in the negative. Wherever the Ottoman dominion has prevailed, civilisation and social improvements have retrograded rather than advanced. We have seen, indeed, that the recently instituted board of health at Constantinople has attributed the exemption of that metropolis from an invasion of the plague for some years past exclusively and entirely to the establishment of lazarettos and quarantine restrictions there; but we must not be too ready to yield our unhesitating assent to this opinion.

It is not necessary to adduce any details to shew that the sanitary condition of such places as Erzeroum and the surrounding villages, of Tunis, Tripoli, &c., has not at all improved of late years, so that they should be less likely to be visited by the pestilence than they may have been hitherto. With respect to Algeria (Chap. VI.)—which seems to have been less frequently the scene of spontaneous epidemics of the plague than any of the other Barbary States, in consequence probably of most of the towns and villages being built upon the slopes of hills, and neither crowded together nor over-peopled—there is good reason to anticipate that, under its present administration, it may become as seldom the theatre of the pestilence as almost any of the countries of Europe.

The answer to the question, proposed in Chap. VII.—*What are the means that should be employed to prevent the development of spontaneous plague?*—must be sufficiently obvious from what has been already said respecting the causes which promote, if they do not induce, the development of the disease in Egypt and elsewhere. M. Villermé has with great ability discussed the general question as to the origin and diffusion of epidemic diseases, and has very satisfactorily shewn that they invariably become less frequent and less destructive in proportion as countries pass from the miseries and degradation of barbarism to the social comforts of civilised life. Dr. Aubert-Roche also has with much care examined this subject, more especially in reference to the plague; and he comes to the same conclusion. In all times, and in all places, this disease has disappeared before civilisation; it has returned with a country's decline and barbarism. Everywhere the same causes have produced the same effects.

### Second Part.

Chap. I.—*Has the plague always exhibited the principal characters of epidemic diseases, when it has raged with violence in Africa, Asia, or Europe?*

The characteristic features of epidemic diseases are these:—1. They generally manifest in their progress three distinct periods, of commencement, persistance or status, and decline. These periods often display neither the same symptoms, the same lesions, nor the same gravity. 2. During the prevalence of an epidemic, other diseases are less numerous than usual, and they receive the stamp or impression of the prevailing affection. 3.

When an epidemic disease prevails, even those persons who retain their health generally feel its morbific influence more or less. 4. Epidemic diseases not unfrequently return and cease at the same season (of the year); and they have usually about the same duration. 5. An epidemic disease is often preceded by other affections, more or less severe and more or less widely diffused; these seem to be in some way its precursors.

Now the plague exhibits each and all of these features in a striking manner. Its severity or malignancy is usually most intense on its outbreak, and for the first few weeks afterwards. Pugnet says that, towards the end of the epidemic at Cairo in the year 1800, almost every patient recovered notwithstanding the most opposite methods of treatment, whereas very few indeed recovered upon its first outbreak.* Not to accumulate authorities, we may state that Clot-Bey remarks that, "when an epidemic commences, almost all who are attacked with it perish. During the first period, death occurs within 24 or 48 hours after the invasion; in the second, on the 4th or 5th day, or it may be not till the 14th or 20th. There are scarcely any fatal cases in the third period;" the pestilence having by this time lost its malignancy.†

It must be obvious from this circumstance, how cautious medical men should be in estimating the value of any remedial means in the treatment of such a disease as the plague, and how important it is to pay great attention to the period of the epidemic visitation when these means have been employed. This is a great practical truth, which is far too little attended to in the present day. "At the commencement of the epidemic (1841)," says Dr. Penay, surgeon-major of a cavalry regiment in the Egyptian army, "I lost almost every patient, in spite of my best exertions. Subsequently, several got well without my being able to determine what line of treatment seemed to be of decided benefit. During the decline of the epidemic, nearly all my patients recovered, and the greater number without any other remedy except local applications to the bubos and carbuncles." The following extract from a report of M. Masserano, one of the members of the Egyptian council of health, is highly illustrative of the same subject.

"While the plague was at its height, almost all the persons who were attacked sunk at the end of four and twenty hours; and such was the violence of the epidemy in some of these cases, that the patients died suddenly while engaged in their employments, as if they had been struck with lightning. The pestilential

---

* *Memoire sur les fievres de mauvais caractere du Levant et des Antilles.* Paris, 1804.

† The following observations of Sydenham may be aptly quoted here:—

*Observare insuper est quod, sicuti epidemicorum quilibet in subjecto particulari suas habet periodos (augmenti scilicet, status, et declinationis) ita etiam constitutio generalis quæcunque, quæ huic alterive morbo epidemice producendo favet, pro ratione temporis quo dominatur, suas etiam periodos habet, quatenus scilicet indies magis et magis epidemice grassatur, donec* ακμην *attigerit suam, atque exinde iisdem fere gradibus decrescat, donec tandem penitus exoluerit, alteri constitutioni locum cedens. Symptomatum enim quod attinet vehementiam, atrociora sunt omnia ubi primum se ostendit; quæ quidem paulatim mitescunt, et in constitutionis catastrophe tam sunt benigna atque* ευφορητα *quam patitur morbi natura in quo fundantur.*—Observ. Med., sect. iv.

characters in the middle, and towards the end, of the epidemy were much less intense. The acute cerebral congestions and complete state of prostration were no longer observed; and petechiæ were of rare occurrence. The sick were distressed with restlessness and weariness; exhaustion and headache threw them into a state of stupor. They experienced more or less severe glandular pains, shooting uneasiness in those parts where bubos were expected to appear: these bubos passed readily into suppuration. When the epidemy approached its close, I saw many persons attacked with bubos, without discontinuing their occupations. Two of my servants, among others, were attacked with the disease in a mild form; they pursued their employment without saying any thing to me. At the time when the disease was most intense, we remarked that, out of 22 persons attacked, 10 died; whereas, towards the end, out of 60 seizures only two proved fatal."

What has now been said will abundantly show how truly the plague exhibits the *first* of the characteristic qualities of Epidemic Diseases. We proceed to examine the *second* one which we enumerated; viz. how far are other diseases, that may exist during the prevalence of the pestilence, influenced and modified by it. Diemerbrock, writing of the plague at Nimeguen in 1635-6, uses these words; *vix ullus morbus peste incomitatus fuit*. Pugnet says that "the plague stamps with its own peculiar character all other co-existing diseases." A proof and, at the same time, an effect of this decided influence of the pestilential constitution upon intercurrent diseases is the circumstance that these resume their own proper physiognomy, (a remark made three hundred years ago by Prosper Alpinus*), as soon as the pestilence subsides. It would be easy to multiply authorities upon this subject, if it were necessary.†

All medical men, who have had an opportunity of studying the plague in Egypt or elsewhere, have remarked that, during the prevalence of an epidemic, those persons, who have already had an attack of the disease, usually feel pain or uneasiness in the scars of their old bubos and carbuncles, without their general health being much affected; and moreover that all those, who have escaped the disease and remain tolerably well, have still nevertheless experienced a certain feeling of *malaise*;—and even a slight degree of tenderness of the lymphatic glands in the groins and axillæ. This is the case equally with those who are in strict quarantine, or who enjoy free pratique. Dr. Delong, in his account of the Epidemic at Cairo in 1841, observes that, it may be fairly said that the entire population had the plague in its first and mildest degree. So impressionable and sensitive, are those, who have once had the plague, to a pestiferous condition of the atmosphere that, it has been supposed, they can generally predict the approach of the disease by the shooting pains and uneasiness in their old bubonic and carbuncular cicatrices. It is possible that in this way we may find out if a pestilential constitution (of atmosphere) exists, or is impending.

Does the plague exhibit the *fourth* character assigned to epidemic mala-

---

* *Medicina Ægyptonem.* Lib. 1, cap. 16.

† Sydenham has remarked, in his description of the plague of London, that the ordinary endemic fevers of a country are apt to retain, for a season or two, after a severe attack of the pestilence, some of its peculiar and characteristic features or symptoms; *pestilenti aeris diathesi etiamnum ex parte perseverante, nec dum in aliam salubriorem penitus immutatâ.*

dies? viz. that of having in general nearly the same duration in different countries, and of appearing and disappearing at epochs which may be determined beforehand?

M. Levison, the Russian Vice-Consul at Alexandria, has drawn up the following statement from the data supplied to him by the Cheik Ibrahim-Bassi:

"The most intense pestilential epidemics in Egypt are those which, commencing *sourdement* in November, have reached their acme about the end of February or during March. On the other hand, those, which have not displayed great violence, have always made their appearance in the course of this last month. In the month of June, both one and the other have often ceased.

The malignant plagues of Egypt have usually lasted about four months, whereas the milder ones have in general not exceeded two months, or two months and a half."

It is a remark as old as the time of Prosper Alpinus, and one which is amply confirmed by the observations of subsequent writers, that the disease in that country almost invariably ceases in the month of June.

At Constantinople, epidemic plague habitually begins in the first or second week of July—during the great summer heats and the prevalence of southerly winds and thick fogs—after or before the arrival of the convoy of merchant ships from Egypt and other places, and usually ceases towards the end of the year. The great plague of 1812, which had been mild up to the end of August, became very malignant in September, carrying off in little more than three months no fewer than 160,000 persons. It entirely ceased by the end of December. At Smyrna, the pestilence generally reaches its height in May, and ceases about the middle of August.

The *fifth* characteristic feature of epidemic diseases—that of being usually preceded by certain precursory maladies—belongs without doubt to the plague. Its outbreaks have been repeatedly observed to be preceded by bad forms of intermittent and continued fevers. "During the winter of 1816-17, there prevailed in this place," writes M. Berbrugger the learned librarian of Algiers, "a very fatal epidemic that was termed in the bills of health a malignant fever. This has been remarked to be a usual precursory sign of an outbreak of the plague itself, when this reappears after a long interval of time." Many analogous circumstances might be quoted. That Typhus not unfrequently precedes, and coexists with, the regular plague is admitted by most of the medical men who have resided for some years in Egypt. Dr. Delong, who lives at Cairo, has made the remark of the epidemic of 1841 that the plague often commenced under an intermittent form, intermittents having been prevalent for some time; and that quinine occasionally seemed to arrest the progress of the malady.

Such being the case respecting the frequent precedence of other epidemic affections to the outbreaks of the plague, it must be obvious that medical men may readily fall into a seeming error, if they happen to be consulted respecting the nature of an existing disease, before the proper pestilence has fairly manifested itself. Hence then the necessity of their giving a guarded opinion, whenever there are grounds to believe that a pestiferous state of the atmosphere is impending. Moveover, at the commencement of an epidemic, there are neither bubos, carbuncles, nor petechiæ in most of the cases. Gaetani-Bey has pointed out a very useful diagnostic sign to direct the medical observer under such circumstances. The lymphatic glands, internal as well as external, should be most attentively examined; and if the patient has died of the plague, one or more of these glands will invariably be found to be enlarged and more vascular than usual. The dissections, made at Abouzabel in 1835 by the medical men who did not then know the opinion of Gaetani-Bey, amply confirmed the justice of his remark.

The facts and observations, adduced in this chapter, lead distinctly to the *conclusion* that " the plague combines in a very marked degree the principal characters of epidemic diseases."

We shall now briefly look at the causes of pestilential plague considered exclusively in this point of view. These causes, like those of all epidemic diseases, are of two kinds. The first relate to the soil and the atmosphere; the second to the physical and moral condition of the inhabitants.

When Dupuytren enquired of the young Egyptian students, who had been brought by Clot-Bey to Paris for medical education, what was the opinion of the most enlightened men in Egypt respecting the origin of the Plague, the answer they gave was, "la peste vient de la terre." All that is conveyed by such an expression is merely that a humid and marshy soil, more or less covered with decaying vegetable and animal matters, is a powerful cause of the alteration of the atmosphere, and consequently of the disease. Now nothing can better serve to shew the importance of the conditions of the soil, in reference to the production of the plague, than the comparing together of two localities in the same country, inhabited by the same people, and governed by the same laws and customs, in one of which the disease is endemic, while the other remains entirely exempt from its attacks, even although the infected may die within its walls.

" Fayoum is elevated above the level of the sea: Damietta borders upon the shore. At Damietta, the air is hot and damp; at Fayoum, it is hot, but dry. Fayoum is free from marshes; Damietta is surrounded with ponds of fresh and salt water. While at Damietta the cemeteries are in the town itself; at Fayoum, they are at a distance from the dwellings. Here, the water, although not very pure, may be drunk without inconvenience, owing to the quantity of nitre it contains; at Damietta, the fresh water is either mixed with sea-water, or it is rendered impure by excrementitious products, and by animal and vegetable matter in a state of putrefaction. Fayoum is surrounded by the desert of Lybia; Damietta is enclosed by rice-fields, and situated in front of the pestiferous Delta."

Great atmospheric vicissitudes have also a decided influence on the development and progress of epidemic plague. Larrey, Pugnet, and all other physicians who have seen the disease in Egypt, agree that its attacks are more frequent, and its mortality greater, when the air is warm and moist, and when the weather has been stormy. At Constantinople, the same causes produce the same effects. We shall not enlarge upon this subject; but only add that it has been too generally supposed that it is to some causes, either actually or recently existing, in the condition of the soil, the atmosphere, or the kind of food, that we must refer the morbific effects observed. And yet, as remarked with his accustomed sagacity by Baron A. Humboldt, the most favourable cause for the development of epidemic diseases is to be found in a uniform and long-continued type of meteorological phenomena. For example, in the case of the plague, it is after a lengthened duration of the same temperature and of the same winds that the pestilence, in an epidemic form, has been observed to break out in Egypt, Syria, and at Constantinople. It may be readily believed that, when a population has lived for a length of time in the same conditions of climate, atmosphere, alimentation, &c., the system of each individual becomes profoundly modified in the same manner, and may be disposed to receive, or even to develope spontaneously, the same disease. Perhaps it is in this way that we may account for what has been very positively asserted to be the case by some authors, but denied by others, viz. that persons, who have been long exposed to the same physical influences, may become affected with the same disease at a given period, although they are then far distant from each other.

The action of epidemic diseases is observed to vary much in point of degree or intensity in different races of mankind, when exposed to the same morbific influences. No fact has been more clearly proved than the very peculiar predisposition of negroes to contract the plague. To Dr. Aubert-Roche we are indebted for the following table of the relative mortality in the different races, during the great plague at Alexandria in 1835:

| | | | | | |
|---|---|---|---|---|---|
| Negroes and Nubians lost . . | 1528 | out of 1800 | = | 84 | per cent. |
| Malays . . . . . . . . . | 367 | „ 600 | = | 61 | „ |
| Arabs, not soldiers . . . . | 10,936 | „ 20,000 | = | 55 | „ |

The Negroes, Nubians, and Arabs were all living in nearly the same hygienic conditions, and were all in free pratique. With respect to the other residents in Alexandria, our conclusions must be more uncertain, in consequence of the very great difference in point of hygienic condition, isolation, &c., enjoyed by different classes of the population. Here, however, are Dr. Roche's calculations:—

| | | | | | |
|---|---|---|---|---|---|
| Greeks . . . . . . . . . . . lost | 257 | in 1800 | = | 14 | per cent. |
| Jews, Armenians, and Copts . . | 482 | „ 4000 | = | 12 | „ |
| Turks . . . . . . . . . . . | 678 | „ 6000 | = | 11 | „ |
| Italians and others from the South of Europe . . . . . . | 118 | „ 1600 | = | 7 | „ |
| French, English, Russians & Germans | 52 | „ 1000 | = | 5 | „ |

These figures carry with them their own signification. It is scarcely necessary to say that the liability to the attacks of the pestilence among all classes of the population, native or stranger, is almost uniformly observed to be inversely proportionate to their cleanliness, good living, and general comfort. An instructive illustration of the truth of this is afforded by Dr. Roche.

"On the banks of the canal, which leads from Alexandria to the Nile, lies a property belonging to the Greek consul, M. Tortizza, who received it as a present from the viceroy. The fellahs who work upon this property, being better treated and better fed than the fellahs of the surrounding villages, only lost, during the epidemic of 1835, 12 out of 400; while their neighbours, placed in the same conditions with respect to atmospheric influences and free communications, lost one half of their number."

Having most satisfactorily shewn that the Plague must be placed in the first rank of epidemic diseases, M. Prus makes the following general reflections upon the subject:—

"The epidemicity of the plague is in truth the fundamental fact of its history, that which most merits the attention of the physician, and which can alone make him comprehend a number of points which, without taking it into account, remain in complete obscurity. The certainty that the plague is a disease which is epidemic in a marked degree, will suggest another consideration to the mind of the physician. It will furnish him with the means sometimes of preventing, and always of diminishing, the ravages of the pestilence. If the existence of epidemic *foci* of plague is satisfactorily demonstrated, things will not affect in the same manner those who remain in or who come into these *foci*, as they do those who are placed, or who remove themselves, beyond their influence.

"Every person remaining in an epidemic focus of plague is exposed to contract this disease. Numerous and authentic facts, observed in Egypt during the years 1835 and 1841, have proved that the most complete isolation and the most severe quarantine do not always preserve those who submit to them. The same remark had been made in as positive a manner at Marseilles and at Toulon, at the time of the plague of 1720.

It requires sometimes but a very short period to be passed in an epidemic focus to become affected with the plague. The professors of the school of medicine at Abouzabel,—which, in 1835, was not attacked by the epidemic influence for more than a month after the capital was ravaged by the disease,—have seen inhabitants of that place, who have only remained a few hours at Cairo, return infected.

"Now, what will happen to persons in health, or already affected with the plague, who shall remove from or be taken beyond the epidemic focus? Before answering this question, we must receive it as a fact recognised by science that, in the most wide-spread and severe pestilential epidemics, experience has shewn that all the localities of the same country have not been subject at the same period to the epidemic influence. It has been stated a hundred times that, by the side of a town ravaged by the plague, other towns in free communication with it continued exempt from the disease. Nay more, plague-patients out of infected towns have come either to die or to be cured in localities where the epidemic influence did not prevail, without the disease having spread. We shall find numerous examples in support of these two propositions, both in the works of modern writers on the plague, and in the documents annexed to this report. Observation has also taught us that it is often tolerably easy to determine the limits of the epidemic focus. This may be circumscribed within the limits of a single town, as Pagnet remarked at Damietta at the time of the plague of 1799, or as was seen at London in 1665, notwithstanding that in both instances the communication with the neighbouring towns remained perfectly free.

"This being established, we may assert, in answer to the question stated above, that, when a population is struck with a pestilential epidemic, persons, whose duties and interests do not require them to remain in the midst of the epidemic focus, will escape the danger by withdrawing from the infected district.

"In 1835, when the epidemic constitution prevailed at Cairo, Gaetani-Bey advised that the 22,000 of the soldiers on active service, composing the garrison, should be sent some leagues from the city, and be encamped under tents in a dry and airy situation, leaving only 2000 invalids for the service of the city. The plague did not commit any ravages among the active troops, whereas it raged among the 2000 invalids as it did among the rest of the population."

Some time before this, Clot-Bey had given a similar advice respecting the fleet which was in the harbour of Alexandria. Although put and kept in severe quarantine, the pestilence made its appearance on board some of the ships, while they remained exposed to the epidemic influence. Not a single case occurred, when the fleet was withdrawn from the focus.

In 1813, Sir Thomas Maitland the Governor of Malta, finding it impossible, in spite of the most severe measures, to extinguish the plague which then prevailed at La Valetta, took the resolution to have barracks constructed fairly out of the city, and obliged the population forthwith to occupy them. From that moment the plague entirely ceased.

Dr. Masserano has related a similar instance with respect to his regiment in garrison at Damietta in 1841; removal to a healthy spot at once put a stop to the disease.

The enlightened portion of the inhabitants of Cairo and Alexandria has already begun to discover the inutility of quarantine within their own dwellings, and now trusts for safety only to removal beyond the sphere of infection. The Persians have long acted upon this principle, and have no doubt found its advantage.*

Removal from an epidemic focus has been found to be most useful, not only in preserving the unattacked from the pestilence, but also in promoting the recovery of those who have caught it. On this latter point, Dr. Delong observes in reference to the plague of 1841:

"When I had the good fortune to be called at the onset of the disease, I instantly ordered a change of abode; whenever it was possible, I caused my patients to be removed to situations that were elevated, dry and airy. The disease then almost always assumed a more favourable appearance, and the morbid phenomena were found less to resist the combined action of nature and of a sound method of treatment."

A similar remark was made by the medical men at Abouzabel in 1835, and it has been repeated by M. Penay in the history of his patients in 1841. The *conclusion*, to be drawn from the numerous facts and observations that have been adduced in this chapter, is surely that,

"Whenever the plague has raged with violence in Africa, Asia and Europe, it has always exhibited the principal characters of epidemic diseases."

CHAP. II.—*What are the differential characters between Epidemic and Sporadic Plague?*

The medical men residing in Egypt, as well as those at Smyrna and Constantinople, agree, almost without exception, that the sporadic form of the disease is not transmissible;† whereas, with respect to the epidemic

---

* Lacheze, *Memoire sur la peste en Perse.*

† The testimony of Dr. Laidlaw, who has resided so long in Egypt, and seen so much of the disease, is very strong upon this point:

"I have no hesitation whatever," says he, "in expressing my decided conviction that, unless the state of the atmosphere is favourable to the spread of the disorder, as is undoubtedly the case during the epidemic, there is no danger whatever from these causes, that they were purely accidental, and that it is impossible to produce by them the spread of the disorder. I have never seen a case of plague occurring sporadically where any person about the patient or in contact with him was attacked; and I cannot find any one that has *seen* one, although it is talked of among the Levantines as a common occurrence."—*Dr. Bowring's Observations.*

form, many of them hold the opposite opinion. This is, indeed, just what might have been expected, for it is in exact accordance with the views entertained by most observers on other analogous diseases (Dysentery for example), which occur at one time sporadically, and at another time epidemically: in the former case, the malady is not transmissible; in the latter, it is often so in a very high degree.

If we admit the accuracy of this opinion, it will be obvious how important it must often be for a medical man to determine if a case of plague be simply sporadic, or if it be connected with a pestiferous constitution of the atmosphere. As a matter of course, this point cannot be fairly determined by a mere summary or off-hand enquiry; things must be carefully watched for some time, before a decided opinion is given. If the disease be limited to a few isolated cases, and if these occur only in the localities where the pestilence arises spontaneously, there will be reason to believe that its type is merely sporadic. The disease too is usually less malignant in this than in the epidemic form. The sporadic plague does not exhibit in its course the three regular periods of commencement, persistance, and decline; nor is it preceded by other epidemic disorders. Moreover, other maladies are not less numerous than usual, nor do they display anything of the prevailing pestilential stamp or impression; persons in health do not experience the effects of an atmospheric influence acting in an especial manner on the lymphatic system; and lastly, whereas the epidemic form of the disease commences between November and February, and ceases about the end of June, the sporadic form is observed in every month of the year. The *conclusion* is therefore very fair and obvious that "Sporadic differs in some very important respects from Epidemic plague."

Chap. III.—*Does the plague extend, like most epidemic diseases, by the migration of certain atmospheric influences, and independently of the agency of the sick who are affected with it?*

Whoever examines with attention and candour the histories of many epidemics of the plague, can scarcely fail to observe that the disease has often broke out about the same time in a number of different localities, distant from and having no intercourse whatsoever with each other. Always originating in unhealthy spots under the influence of those causes which have been already mentioned, the epidemic pestilence may be either confined within the circuit of a single town or city, although this remains in free communication with the surrounding district, as occurred at Damietta in the year 1799; or else it may become diffused over a number of countries, as in the formidable epidemics of 542 and 1348. It is scarcely necessary to adduce further instances to prove that the localities and districts, immediately adjoining to an infected spot, often remain quite exempt from the disease. Not unfrequently several towns are attacked about the same time, the intermediate villages remaining quite free; at other times, the pestilence advances in a more regular manner, attacking a number of places "de proche en proche" and in succession.

Clot-Bey and Dr. Roche are of opinion that the plague may traverse seas, and pass from one continent to another—from Alexandria to Marseilles, for example—through the medium of the atmosphere alone. On the other hand, that it may meet with unsurmountable obstructions not far from the spot where it has arisen, would seem to be the case from the well known fact that the plague of Lower Egypt never passes beyond the first Cataract of the Nile. In some malignant epidemics, indeed, the disease spreads into districts that are very generally spared. This has been observed at certain periods in Upper Egypt and the Hedjaz.

It appears that the plague never extends to a great elevation above the level of the sea. There is a village about five leagues from Constantinople, situated on the mountain of Alem-Daghe at an elevation of about 500 *metres*, where the pestilence has never been known to exist: it serves, indeed, as a place of retreat to the inhabitants of the Turkish capital during the prevalence of an epidemic. On the same mountain, but at a less elevation, there is another village that by no means enjoys the exemption of the first. There is a spot in Malta to which the plague has never reached; from this circumstance it has received the name of safi (pure). According to the testimony of Desgenettes and Clot-Bey, the Citadel of Cairo, which stands upon a lofty eminence, has uniformly escaped during the worst epidemics that ever raged in that city.

A most important question here suggests itself to our consideration; viz: *When a pestilential epidemic prevails in a place, how many cases of the disease are to be attributed to the influence of the epidemic constitution, and how many either to the absorption of the miasms emanating from the sick, or to direct or indirect contact with them?* As may be imagined, the solution of this problem is attended with many difficulties. Dr. Lacheze, when physician of the hospital at Cairo in 1835, endeavoured to determine the point. According to the observations of this gentleman, not more than one person in 400 of those who were entirely isolated or in quarantine was attacked; whereas the pestilence of that year carried off no fewer than one in three of the general population, that remained in free pratique. Without disputing the accuracy of the figures given by this gentlemen, it is the opinion of many able observers that they should be interpreted very differently from what M. Lacheze has done; at all events, it must never be forgotten that the very persons, who put themselves in quarantine during the prevalence of an epidemic, are precisely those who enjoy the greatest comforts of life and pay most attention to a hygienic regimen. The Commissioners were therefore desirous of obtaining if possible some less objectionable data for comparison, and it occurred to them that the best plan would be to ascertain the results in some large public establishment, either at Cairo or Alexandria, the inmates of which were living in very nearly the same conditions as the rest of the population out of doors. For this purpose, the Arsenal at Alexandria, which always contained 6000 workmen at the least, was selected for observation. Now, what has been the result of their enquiries? The words of the report are these:

"In this establishment, no attack could be attributed to an accumulation of pestilential miasms. Such an accumulation never existed; for, whenever an invalid was discovered to be infected, he was instantly taken into an hospital situated without the arsenal. Neither could contact with those infected be regarded as the cause; since, whether from the invalids having been removed at the beginning of the disease, or from quite a different cause, the neighbours of those who were seized with plague, as well as those who had touched them, were never attacked with the disease. The number of workmen at the arsenal, removed to the hospital on account of plague, gives us then that of the cases attributable to the epidemic, apart from any other, agency. Now 300 workmen having been attacked with the disease in a total of nearly 6000, we may fairly believe that the epidemic influence alone acted upon one individual in every twenty. This proportion is without doubt very different from that pointed out by M. Lachèze; but it also varies very considerably from that furnished by the population in free pratique; for this at Cairo and Alexandria, as we have already seen, lost one in every three. Are we to believe, with Clot-Bey, that the difference of hygienic condition completely accounts for these facts, and that, if the mortality among the workmen in the hospital did not amount to one in every three of their number, this was solely owing to their having been kept cleaner and better fed than the rest of the working population at Cairo and Alexandria?

"While we readily recognize and publicly admit the very great power of hygiene in preventing and moderating the ravages of the plague, we must at the same time distinctly state that the deductions drawn by Clot-Bey appear to us to exceed what the facts warrant.

"Neither can we assent to his final conclusion, that all cases of plague should be attributed to epidemic influence alone. We must reject this conclusion, on the one hand, because it does not appear to us to be based upon positive and satisfactory proofs; and on the other hand, because, if it were rashly received, it would have the serious inconvenience of checking all enquiry into those causes, which, secondarily, tend to propagate the plague, and increase its fatality. In science, a false explication is less dangerous in that it extends error, than that it impedes the search after truth.

"From the facts and considerations contained in this chapter, we think we are fairly justified in drawing the following *conclusion:*

"The plague, abstractedly from the influence which the infected may exercise, spreads itself after the manner of most epidemic diseases, viz. by the action of general causes."

### Third Part.

We now approach the question of the transmissibility of the plague, away from or beyond, as well as within, epidemic foci—a question, we need scarcely say, of the highest importance and, at the same time, of very difficult solution. The first point that we shall discuss is this:—

### Chap. I.—*Is the plague transmissible by inoculation either of the blood drawn from a vein, or of the pus from a bubo, or of the serosity from the phlyctena of a carbuncle?*

"It is obvious," says M. Prus most justly, "that if the plague be truly a virulent disease (*à virus fixe,* to use the expression of certain writers), the possibility of the inoculation of its virus would approximate it to epidemic contagious diseases; whereas if it does not furnish any principle, liquid or solid, that is susceptible of being inoculated and of producing a virus similar to that which gave it birth, the disease must be withdrawn from the class of diseases that are properly contagious, such as Small-pox, and would approach in this respect the character of Typhus, which is propagated by peculiar miasms, but which gives out no inoculable element."

And here it should be noted as an important fact that, if the diseases that are indubitably contagious—Small-pox, Hydrophobia, Glanders, and Syphilis, for example—all present us with a palpable liquid which contains the poisonous principle, such is certainly not the case with the Plague. Hence medical men have operated, by turns and almost indifferently, with the pus of a bubo, the serosity of a carbuncle, or even with the blood itself of a pest-patient.

The experiments that were made in Egypt by Desgenettes, White, and a few other physicians, about the beginning of the present century, to ascertain the effect of the direct inoculation of the matter taken from plague buboes, are anything but satisfactory or conclusive. We shall therefore not dwell upon them, but at once proceed to notice those which were instituted in 1835 at the Cairo Hospital in the presence of Gaetani-Bey, Clot-Bey, and Drs. Lacheze and Bulard, and which are deserving of all confidence.

Five criminals, who had been condemned to death, were the subjects of the experiments. A lancet, wetted with the blood drawn from a pest-patient, was passed under the epidermis on the inside of the arm of one of these criminals, at two different points. On the third day afterwards, the man was affected with confirmed plague—so, at least, says Dr. Lacheze, who reports the experiment; Clot-Bey thought the case doubtful. Three days subsequently, the man was convalescent.

In three other cases, no effects followed the inoculation of the blood. In two cases, the serosity from a carbuncle, and in one the pus from a bubo, was used for the purpose of inoculation: in none of these cases, was the disease induced.

With respect to the single case, in which the disease (mild indeed) occurred after inoculation with the blood of a pest-patient, it must be kept in mind not only that the man was exposed, as a matter of course, to the epidemic atmospheric influences then existing in Cairo, but also that, for three days before the performance of the experiment, he had been living in a pest-hospital, which was necessarily a focus of pestilential infection.

Clot-Bey inoculated himself, in six different punctures, with the blood of a pest-patient: no constitutional effects followed. A few days subsequently, he inserted some pus from a bubo on the inner part of his left arm: this was followed by a slight indisposition, which he attributed to the absorption of the purulent matter, but which bore no resemblance to the symptoms of plague.

The results of certain trials made by Professor Pruner in 1829, and by Dr. Rossi in 1841, were altogether similar.

The general *conclusion* of the Commissioners upon the important point under consideration is to this effect:

"The results of the inoculation of the blood drawn from the vein of a plague patient, or from the pus of a pestilential bubo, have been equivocal; the inoculation of the serosity taken from the phlyctenæ of a pestilential carbuncle has never given the disease. It is therefore not proved that the plague can be transmitted by inoculation, even under the influence of a pestilential constitution.

"We are not acquainted with any experiments that have been made upon the same subject, at a distance from an epidemic focus.

"It is useless to observe that the study of the effects which might have been obtained from inoculation of the plague, a study so important for the knowledge of the nature of the disease and consequently of its transmissibility, presents, nevertheless, no direct application to the question of quarantine. There can be no fear that the mass of a population will ever allow themselves to be inoculated with the plague."

Chap. II.—*Is the plague observed, in epidemic foci, to be transmissible by immediate and direct contact with the sick ?*

The Arabian physicians, as well as their predecessors, regarded the disease as purely and simply epidemic, and seem therefore not to have troubled themselves in seeking to determine if the disease be communicable from one person to another. We must come down to the middle of the 15th century, the time of Frascatorius, before we meet with any formal exposition of this doctrine. The celebrated physician of Verona recognised three modes in which the plague may be communicated :—1, direct contact with the sick ; 2, the infection or contamination of goods, clothes, &c. ; and 3, diffusion of morbific miasms through the atmosphere. The relative frequency of these three modes was believed to be in the order that they are here enumerated ; the first being supposed to be by far the most common, and the last to be comparatively very rare. These opinions of Frascatorius prevailed almost universally down to the year 1720. In In that year, Chicoyneau, Verney, and Deidier of Montpelier maintained with considerable eclat the doctrine of the non-contagiousness of the plague ; they regarded it as purely epidemic. Their chief argument was, that they had touched the bodies of plague-patients without taking any precautions, and that they had not caught the disease. The opposite and older opinion, however, continued to be very generally held in the schools.

In 1771, Mertens, Orræus and Samoilowitz, who had an opportunity of watching the plague at Moscow, declared their belief that it was propagable only by direct or indirect contact with the infected, and never through the mere medium of the air. Stoll, however—who is characterised by Dr. Prus as the most able observer, after Hippocrates and Sydenham, of epidemic diseases—was not at all satisfied with the prevailing opinions on the subject in question, and pointed out in the following (ironical ?) passage the necessity of re-examining them with care and candour.

"He who would deny," says this truly enlightened physician, "the contagion of the plague, and attribute a very grave disease to an epidemic cause, acting equally upon all, but not producing equally upon all the same effects, and would ascribe it either to the constitution of the year, or to an alteration in the air more fit to produce putrid diseases than in other years, that person, I say, would assert (what would be considered) a paradox. But, at the same time, whatever truth he might utter, and whatever service he might render in the calamitous conjucture, it were well for him to be at a distance. He, who would hold this opinion, might find abundance of arguments, which could not be refuted, in all the author's who have written on the plague, even in those who have defended contagion ; unless, indeed, the love of the marvellous should make him despise or overlook the most simple causes, which he might find at his very feet."

All the medical men who accompanied the French expedition to Egypt, Assalini alone excepted, were of the opinion that the plague is propagated by contact with the infected. For nearly forty years after their return, this opinion has been universally received, and acted upon. It was not till 1835, that a change of sentiment began to be manifested among medical men on this most important subject. In the course of that year, as we have already seen, a number of European physicians had an opportunity of studying the terrible pestilential epidemic that ravaged Egypt. Impressed at first most firmly with the belief of the transmissibility of the disease by contact with the sick, they have all, with scarcely one exception, completely changed their opinion; as, indeed, M.M. Brayer and Cholet, who had observed the epidemics of 1819, 1826, and 1834 at Constantinople, had previously done. The writings of these last-named gentlemen, and subsequently of Clot-Bey and Aubert-Roche, have mainly contributed to effect this very remarkable revolution in medical doctrine. We shall briefly note a few of the most interesting facts, which have been of late years made public.

During the pestilence of 1824, upwards of 30,000 persons died in Cairo, while not more than two or three cases occurred in Alexandria, although the communication between these two cities was constant and uninterrupted. In 1834, on the other hand, the plague broke out and continued in Alexandria for a very considerable time, before it made its appearance at Cairo; and it had existed for fully eight months in the former city, before there was any sign of it in Mansoura and Damietta, although the daily intercourse between these places remained entirely free. Dr. Coch, principal physician of the Egyptian fleet, mentions an interesting fact observed by him in 1835. Ten men had gone from Sakkarah, a populous village, to Cairo, where the plague then existed. On their return home, every one of these men sickened, and died; yet not a single member of their families, who had assiduously waited upon them, took the disease. "Such a fact," it is emphatically added, "was observed hundreds of times during the course of this great epidemic." The same gentleman states that, the Viceroy having ordered that all vessels in which the plague appeared should be subjected to a quarantine of 11 days, the sick were immediately disembarked and carried on shore by the sailors of the fleet; and, although these sailors returned on board and communicated freely with the rest of the crews, not a single case of infection was the result.

We owe the following facts to Dr. Roche. The ports of Suez and Cosseir on the Red Sea draw the chief supply of their provisions, the one from Cairo, the other from Keneh in Upper Egypt. In 1835, the plague broke out at the latter place about the same time that it made its appearance at Cairo. Suez was attacked by the pestilence; but Cosseir remained quite exempt. The first of these places is surrounded by stagnant marshes, a state of things not unlike to what exists in all the towns of the Delta; the second is built upon rocks, and is surrounded by bare arid hills. During this epidemic, Djedda, Yambo, and Moka enjoyed the same immunity as Cosseir, although the sick from Suez and other infected localities often died in the midst of them. Still more convincing is the following statement:—

Every year pilgrims depart from all parts of the country, subject to the laws of Mahomet, to go to Mecca. Caravans from Morocco, Darfour, Egypt, Constantinople, Persia, Asia Minor, and Syria converge at Djedda, at Medina, then at Mecca, the central point. They carry merchandise with them, for this pilgrimage is also a fair. Has the plague ever broken out at the place of meeting of all this population and all this merchandise, which have often, be it remembered, come from places infected by it? No. On the contrary, it is proved that, from time immemorial, the plague has never been seen in Arabia. The epidemic plagues, which desolated a great part of Lower Egypt in 1825 and 1835, had not one victim in

Arabia, notwithstanding the daily and perfectly free communication which existed between these countries. This has also invariably been the case with respect to the pestilential epidemics of Constantinople, Smyrna, or Syria. The Arabian historians pretend that their country owes this immunity to the protection of the Prophet!

Nubia, Sennaar, and Abyssinia, notwithstanding their close connexion with Egypt, are not acquainted with the plague. If it may be said regarding Arabia, Sennaar, and Nubia, that the heat in these places prevents the condensation of pestilential miasms; the same reason cannot be alleged for Abyssinia, which is a temperate country, the thermometer varying from 16 to 25 degrees Cent. above zero. Here the salubrity of the climate alone serves to keep the disease at bay. Abyssinia is a mountainous country with inclined plains, where there are neither marshes nor stagnant waters.*

For the extract which follows we are indebted to the work of Clot-Bey; allusion has been made to it in a former page.

"During the five months that the epidemic of 1835 lasted, MM. Gaëtani, Lachèze, Bulard, and myself at Cairo, MM. Duvigneau, Scisson, Perron, Fischer, at Abouz-Abel, and MM. Rigaud and Aubert, at Alexandria, visited the infected in the hospitals and in private houses. None of us took the least prophylactic precaution. We were in immediate contact with the sick during all the stages of the disease. We received upon our clothes and upon our hands the matter that was rejected by vomiting, the blood of those who were bled, the pus from the thousands of bubos which we opened. More than a hundred dissections were made at Cairo, and we passed whole hours in endeavouring to detect, in the bodies of those who had just expired, the pathological alterations which had hitherto been so little attended to. The same researches were made with equal care at Alexandria.

"Dr. Rigaud is the only one among us who fell a victim to the reigning epidemic.

"It is remarkable that many physicians, who scrupulously avoided all contact with the sick and with suspected objects, were attacked with the plague and died. Of this number are Dr. Mannucchi, sen., Leopold and Lardoni."

The observations made by different medical men, during the subsequent epidemics of 1837 in Syria and of 1841 in Egypt, amply confirm these statements of Dr. Roche and Clot-Bey. M. Granet was the chief medical officer of the troops stationed in the province of Adana (Upper Syria), when the plague broke out there in 1837. He was entrusted with all the sanitary regulations ordered by the governor. At first, it was attempted to arrest the extension of the pestilence by establishing a cordon around the infected spot. This was speedily found to be wholly useless. Upwards of 15 new cases were received every day into the military hospital, in which there were usually from 40 to 60 cases at a time. No precaution to guard against the risk of contagion was employed either by the medical men or by the other attendants of the sick; and yet not a

* Aubert-Roche, *ouvrage cité*, p. 100.

single case of the disease occurred among them, although the epidemic lasted for three months. " How can we believe," says M. Granet after relating these particulars, " that, if the plague was really transmissible by contact with the sick, we should not have had a single case of this transmission?"

The evidence of Dr. Ibrahim, a native physician of Cairo, is highly satisfactory and convincing. He adduces many cases where one member only of a large household was affected with the plague, although the patient had been waited upon by the whole body of the domestics. The case of the wife of Hassan-Pacha, who died on the 35th day of the disease, is more than usually instructive ; she had no fewer than two dozen white and black slaves, two keios, two eunuchs, and four pages, in constant attendance upon her!

Dr. Delong also and M. Euzieres report many cases that occurred under their own immediate notice in Cairo, during the epidemic of 1841, in which the relatives of the sick, who had most assiduously nursed them up to the hour of death, entirely escaped. It seems unnecessary to give the details of any of them. In two cases, one of which proved fatal on the fifth day of the attack, the patients continued to suckle their infants ; the children were not affected. In several instances, the disease attacked those who had sequestrated themselves by the most strict quarantine from all communication with the city; while others of their household, who were less timid, remained intact.

The testimony of Dr. Arnoux, surgeon-major of the 43rd regiment (Egyptian army) stationed in 1841 at Nabaro, of Dr. Dieterich of the 5th at Damietta, of Dr. Penay of the 5th cavalry at Neguille, and of Dr. Chedufau, chief physician of the central military hospital at Cairo during the same year, all serves to prove that the immediate attendants upon plague-patients are, on the whole, very rarely infected ;—always provided due regard be paid to the ordinary hygienic precautions. The last-named gentleman informs us that, in consequence of there being no separate plague-hospital prepared when the pestilence broke out, the sick were received at first into the general hospital along with the other patients. There were no fewer than 182 cases of plague treated in this hospital ; and, although the number of the ordinary inmates and attendants during this period amounted to 2,000, no instance of infection could be directly made out. None of the " officiers de santé," consisting of 92 Europeans and 300 Arabs, remained in quarantine, but waited upon the sick without taking any precautions. Only three of the Europeans were attacked ; and, of these, two recovered. Three also of the Arabs suffered ; they all died. M. Chedufau himself, besides his hospital duties, treated many cases of plague in the town, opening bubos, dressing carbuncles, and executing all the necessary medical duties to his patients. He performed, moreover, 17 *post-mortem* examinations. During the whole of the epidemic, he was in continual intercourse with the members of his own family ; and yet, although no preservative means were employed, neither he nor any of his household suffered.

The experience of Dr. Perron, the director of the medical school at Cairo, confirms in every respect the truth of these statements. Not one of the professors or pupils, who were in daily attendance upon the sick, was infected.*

After relating numerous other facts and statements, all bearing upon the question proposed at the beginning of this chapter, the *conclusion* is at length arrived at, that,

"On the one hand, immediate contact with thousands of plague-patients has not been followed by any dangerous consequences to those who have been exposed to it in the open air or in well-ventilated chambers; and on the other, that there is not a single fact which indisputably proves the transmissibility of the plague by mere contact with the sick."

Chap. III.—*Is the plague transmissible by contact with the clothes or effects of the sick, in localities which are, or have been, recently exposed to the epidemic influence?*

After the plague of 1835 at Cairo, the clothing, effects, &c. of upwards of 50,000 plague-patients, who had been carried off by the pestilence, were sold in the public bazaars, without communicating, as far as is known, the disease in a single instance. More than 600 houses remained tenantless for several months after this frightful epidemic; they were then ordered to be visited, and an inventory taken of their contents. Not one of the persons engaged in this service fell sick.

Three thousand plague patients were received into the large hospital of Esbequiè during the year 1835. On the cessation of the pestilence, the ordinary class of patients was admitted and put into the very same beds, which had been occupied by those who had died of the plague. The sheets indeed were changed, but the coverlets, which had never been subjected to any process of disinfection nor even freely exposed to the air, were used without alarm. No case of infection ensued. These facts were made known in 1840 by Clot-Bey, and have not since been disputed by anyone.

Dr. Brayer informs us that it is a fact perfectly well known in Constantinople, that the Jews buy up the clothes, &c., of persons who have died of the plague, however virulent this may have been, for sale at Fit-Bazar, where most of their stores are. No one dreams of using any means of disinfection, If the deaths be numerous, the bazar is full; and *vice versa*. All the poorer classes resort thither for their clothing; and, generally, bundle after bundle is turned over and examined, before a purchase is made. In 1812, the "depouilles" of 150,000 victims of the dreadful pestilence of that year were brought together into that market! One portion was speedily bought by the inhabitants of the city; another portion was forthwith sent away into all the principal towns throughout the Turkish dominions; while what remained unsold was kept in close confined magazines, to be disposed of next year. Notwithstanding this dispersion of infected *fomites* to such a vast extent, nothing was heard of the spread of the pestilence thereby. It deserves also to be noticed that a smaller proportion of the Jews died than the Greeks, who, we may remark, have always had great fear of the contagion of the plague.

---

* We shall afterwards however find it stated that most of the *pharmaciens* in one of the hospitals at Alexandria caught the disease and died, and also that very many of the pupils in the hospitals at Cairo perished.

It would be easy to mention many other instances where the clothing, goods, equipage, &c. of persons affected with the plague have been taken possession of by others, without any injurious results. But this is unnecessary; suffice it to say that, in Egypt, Constantinople, Smyrna, &c. an epidemic of the plague is almost invariably found to subside and cease at a certain period known beforehand, whether any sanitary regulations have been taken to arrest its course or not; and that then the clothes and other property of the victims freely circulate in the bazaars of the place. If these objects communicated the disease to those who handled them, it is quite obvious that it would last much beyond the period at which it disappears with a truly remarkable regularity.

Such are some of the facts that have led most of the medical men resident in the East to believe, that the plague is not transmissible through the medium of *fomites*. Even although we do not go so far as this, it may surely with perfect truth be asserted, that in a vast number of instances, some of them indeed of almost continual recurrence, the pestilence has not been communicated by touching, or even wearing the apparel of those who have died from it, although no means of purification or disinfection had been employed. We are not prepared utterly to deny that the disease has been, and may be, sometimes transmitted in this way;* but surely, after the facts which we have mentioned, we may very fairly withold our credence from many of those histories on record of the plague having arisen in places previously quite healthy, from the introduction of infected objects. Most of the reported facts of this description appear to have been dictated by prejudice and accepted with credulity. Nevertheless, it must be admitted that some medical men, who have had extensive opportunities of observation, still maintain the opposite opinion. Of these, M. Grassi, principal physician of the lazaretto at Alexandria, and who has been in the Egyptian service for upwards of 20 years, is the person whose opinion is entitled to perhaps most consideration. We may state, however,

---

* Clot-Bey himself reports the following narrative:—

"On the 15th of April, 1835, (in presence of MM. Gaetani, Clot, Lacheze, and Bulard) two young criminals, Ibrahim-Assan and Ben-Ali, in perfect health at the time, were placed in beds which had just been left by patients affected with well-marked plague.

"In the night of the 18th, Ibrahim's pulse was slightly affected.

"The following day, he had the plague with bubos and carbuncles; he died on the 23rd.

"On the third day after he was in bed, Ben-Ali also felt the ordinary symptoms of an attack of the plague; but the disease abated, and convalescence began from the fourth day after the appearance of the characteristic symptoms.

"That Ibrahim-Assan died of the plague, after having slept in a bed recently left by an infected person, is a fact. But was it the sheets or other coverings of the bed which gave the disease? This is uncertain. Ibrahim-Assan was in a town where a pestilential epidemic raged; he was in a hospital which had contained and did still contain a great number of plague-patients, and in which several medical pupils and attendants had contracted the disease. It cannot then, in his case, be absolutely asserted that the plague developed itself by contact with contaminated objects, rather than by epidemic influence alone, or by miasmatic infection."

that the accuracy of many of the facts, which he has adduced in favour of his views, has been disputed or denied by Clot-Bey and others.

The general *conclusion,* to which the Commissioners have come, after a laborious investigation of all the particulars, is that

> " Very numerous facts prove that the clothes and effects, belonging to plague patients, have not communicated the disease to persons who have used them, even without any previous purification. The facts, which seem to indicate an opposite result, can only be considered valuable, if they are confirmed by fresh observations made beyond epidemic *foci,* at a distance alike from *foci* of miasmatic infection and from countries where the plague is endemic."

CHAP. IV.—*Is the plague transmissible, in countries where it is endemic or epidemic, by merchandize suspected to contain pestiferous matters ?*

It will be obvious that it must be very difficult to decide this point in places where the pestilence is indigenous, and where it is, therefore, liable to be developed spontaneously at any time ; for what may be attributed by certain persons to handling some description of goods, may be altogether owing to the influence of an endemic cause. The case related by M. Sicard of Marseilles to M. Prus is unsatisfactory in several points of view, and nearly the same thing may be said of all the analogous examples which have been made public. It may, therefore, be very fairly set down that the transmissibility of the plague, under the circumstances mentioned, has at all events not been proved.

CHAP. V.—*Has the plague been observed, in epidemic foci, to be transmitted by the air being charged with pestiferous miasms ?*—in other words, is the plague infectious through the medium of the atmosphere during the prevalence of an epidemic pestilence ? Until of late years, the question of atmospheric *infection* had been altogether superseded by and merged in that of direct and immediate *contagion.* We need scarcely say that it can never be an easy thing to determine with exactitude the infectiousness of any disease, while a pestilential constitution of the atmosphere exists, and when consequently a whole population is exposed to the morbific influence. As we have previously remarked, scarcely a single person escapes *in toto* the effects of the malarious condition of the air ; they are experienced by all to a certain degree during the prevalence of the epidemy.

We shall first enquire if the air of a plague-hospital has seemed to give the disease to those, who had most carefully avoided all contact with infected persons or objects. That great mortality occurred among the *pharmaciens* and attendants in the plague hospitals at Cairo and Alexandria during the pestilence of 1835, notwithstanding the use of all precautionary means to avoid direct contact with the sick, or of any thing belonging to them, cannot be disputed : of 20 pupils, that were sent from Abouzabel to the hospitals of Cairo, 19 caught the disease and died. The question then comes to be, was this mortality owing to the general epidemic influence, to local atmospheric infection, or to immediate contact with the patients ? The best answer to this will probably be found by referring to what took place at Abouzabel itself—only a few miles distant, it will be remembered, from Cairo—when that place was attacked by the pestilence. The barracks of the sick were situated in the open country, at some elevation above the plain, and well ventilated. Not one of the medical inmates or attendants, although in most frequent contact with infected persons and objects, suffered ; and this too, be it remembered, in an epidemic focus. The free ventilation had prevented the formation of " a focus of infection." Dr. Laidlaw, the physician of the general European hospital at Alexandria, remarks most truly that " whenever a number of plague-patients are collected together in one space, they seem to create a pestilential atmosphere, unless free ventilation be employed." Hence the importance of never having many plague patients in one room or ward, and of maintaining a most thorough ventilation through every part of the building, which should always, if possible, be situated in an elevated open position and on a dry foundation, at a distance from stagnant water or any source of malarious exhalations. If these circumstances be not attended to, hospitals are apt to become positive foci of infection, and thus to increase rather than to diminish the general mortality.

That the disease, when it has attacked one of the inmates of a house, is, under certain circumstances, apt to extend to others who are dwelling there, cannot be disputed. Dr. Gràssi tells us that, in 1835, no fewer than 57 deaths occurred in the house of Hingi Osman, the treasurer of the marine at Alexandria. This is only one out of many similar instances that might be quoted. The fair conclusion therefore seems to be, that pestilential miasms or effluvia emanate from the bodies of the sick, and that these powerfully tend to act upon the system of persons whom the epidemic influence has already made liable to the disease. Almost all the resident medical men in Egypt at the present time, who believe in the transmissibility of the plague, are of opinion that the transmission is effected in this way. Dr. Grassi is the only one who maintains that the disease is communicated by direct or indirect contact with the sick, without any intervention of atmospheric agency.

Remaining long in the chamber of the infected is the thing most to be avoided by the medical and other attendants. What Dr. Rigaud said to his friend M. Lesseps, the French Consul-general, who visited him constantly to the last moment of his life—" Do come and see me twenty times a day, but don't remain more than five minutes at a time in my room"—abundantly shews what his sentiments were upon this point.

By a singular perversion, the common practice in the East is to keep a patient in a close and confined apartment, with as imperfect a current of air through it as possible. What wonder then that a disease like plague spreads, wherever it makes its appearance: every sick chamber becomes a new focus of infection ! There cannot, therefore, be a reasonable doubt that the diffusion and mortality of the pestilence are powerfully promoted by the contamination of the atmosphere with morbific emanations from the bodies of the infected.

Sometimes it happens that when a locality, which has contained a number of plague-patients, comes to be occupied by other persons (who may use nevertheless every possible precaution to avoid all contact with suspected objects), the disease re-appears to a greater extent among the new residents than can fairly be attributed to the sole agency of the epidemic influence which may be then existing.

In 1834 in the month of June, during the insurrection which broke out in Judea, the insurgents pillaged and sacked Jerusalem. A number of Roman Catholics took refuge in the convent of St. Saviour in this city.

" At the end of ten or twelve days of close confinement, I remarked," says M. Delong, " cases of plague among this distressed population, huddled together in their dormitories, upon and under the stairs, in the courts and other chambers of this vast building. After twenty-five days of expectation, Ibrahim Pacha at length arrived, and the city was relieved. The holy Fathers, full of alarm, hastened to clear their dwelling of all this mass of people, and shut themselves up in most strict quarantine. What happened? Of all those who left the convent, three only died four or five days afterwards. But, out of 63 priests who thought to save themselves by isolation, no fewer than 22 died."

" Now," continues M. Delong, " what part did infection play in this instance? I will not positively decide. It seems however clear to me, that we should, in all similar circumstances, disperse the infected into different quarters, instead of shutting them up with others, as yet intact, in a confined locality where sanitory regulations have not always been attended to."

What occurred in the musical academy at Kanke in 1835 is still more deserving of attention :—

The plague having broken out in this school, although it was kept in the strictest quarantine, the pupils were sent into the desert, where they continued for upwards of a month. In the mean time, all the rooms were well cleansed and purified; and no person had remained in the building. Not one case of plague occurred in the desert: but no sooner had the boys returned to their old quarters, than several were taken ill; and each day several fresh cases were reported. Again were the boys sent into the desert; and again the disease ceased to spread. While they continued in the desert, 15 soldiers were employed to go daily to the village, where the plague was raging, for provisions; but none of these men caught the disease themselves, or gave it to the boys.

During this disastrous year, many striking circumstances similar to those now mentioned occurred in the military barracks, all of which had been put into strict quarantine. Although every attention was paid to cleanliness, they seemed to remain foci of infection whenever the disease had once shewn itself in them.

It will be obvious that the facts now mentioned, coupled with similar ones observed in epidemic foci and more especially on board-ship, must receive new and very useful applications in considering the question of Quarantine.

Admitting that morbific miasms are given off from the bodies of plague-patients, and thus render the surrounding atmosphere pestiferous, we have next to enquire whether these miasms are exhaled from the lungs, or from the surface of the body of the sick, or from both; and also when they prove infectious, whether they are (most probably) absorbed by the skin, the lungs, or in the way of deglutition. There can be very little doubt in the present day that very many cases, where the disease has appeared to be the result of direct contact with a patient or with any thing he has used, may fairly be regarded as examples of infection through the medium of the atmosphere. Dr. Brayer has put this point very forcibly :

" A person finds himself affected with plague, although he has never left the house; straightway he tries to bring the mind where he was the night before, two or three days ago, the objects he touched, and so-forth. If he has not been out of doors for one or two weeks, owing to indisposition, it matters little; he was out three weeks, a month ago; and as the virus may be retained for months, nay, for years, it is not surprising that the disease should have shewn itself. He will not for a moment consider that the skin is guarded by the double covering of the epidermis and of the clothes which he wears, and that therefore the virus can only with the greatest difficulty be transmitted from the exterior to the interior. As to pulmonary absorption, that is never spoken of. It is a received opinion among the Franks that the air is not, never has been, and never can be the vehicle of the plague. They refuse to believe that, in seasons of the plague, every individual breathes an air more or less deleterious, that the person who sees or touches an invalid is in the atmosphere of the invalid, and that when, by means of the air, a deleterious principle is conveyed into the minutest bronchial ramifications, and by the act of deglutition into the gastric passages, there is then much more than mere contact; for there is a real penetration in the first case; and, in the second, there is digestion and interior absorption. Infection therefore exists, in the strongest sense of the term; and the disease which it occasions is more or less severe, according to the quantity of miasmata introduced into the system, their intensity, and the individual predispositions or susceptibilities of those who have absorbed them."

The Academic Society of Marseilles, in the report which was unanimously adopted in August 1845, expressly recognises the following two propositions:—

"1. Writers, the most at variance in all that concerns the general history of the plague, are nearly unanimous in asserting that the simple contact of one individual with another is one of the modes of transmission the least favorable to the propagation of the pestilence.

"2. A lengthened stay in the atmosphere of the sick, and, above all, exposure to the pestilential miasms which contaminated objects exhale are highly dangerous."

The only difference, says M. Prus, which, upon this truly important point of doctrine, exists between the Academical Society of Marseilles and the Commissioners, may be thus summed up:—

The Academic Society asserts that the simple contact of individuals is one of the modes of transmission least favorable to the propagation of the plague.

The Commissioners think that no well-authenticated fact establishes the reality of such transmission. They are moreover not acquainted with any facts to authorise them to believe, with the Academical Society, in the dangers of miasms from contaminated objects.

The Academical Society and the Commissioners equally acknowledge that a lengthened stay in the atmosphere of infected persons, or, in other words, infection by pestilential miasms is that which is most to be feared.

Dr. Mead who, at the time of the plague at Marseilles in 1720, was ordered by the English government to draw up instructions for preventing, if possible, the introduction of the pestilence in England, and to arrest it if it was introduced, has much insisted upon the utility and necessity of promptly removing the sick from the seat of the infection, and transporting them to some distance.*

---

* R. Mead, M.D. *A short Discourse concerning Pestilential Contagion.*—London, 1720; and *De Peste Liber*, 1723.

He has mentioned as worthy of imitation the course pursued at Rome, during the plague of 1657, by Cardinal Gastaldy, at that time invested with full power to take every sanitory measure which he judged proper.

The Cardinal prohibited any infected person, and even any person in health who was suspected, to remain in their houses. They were promptly taken to the hospital, built on the island which divides the Tiber. With respect to those who had occupied the same house, they were placed in other hospitals near the city, from whence they were removed into the island if the disease shewed itself. During this time, the Cardinal was very careful to have all the furniture taken out of the infected houses, exposed in the open air, and the apartments left open, in order to purify them.

By these means the Cardinal, in two months, caused the plague to cease, after it had raged at Rome for two years.

But that which deserves most attention, adds Dr. Mead, is, that, before these regulations, it was constantly observed that the disease rarely appeared in a house without attacking all its inhabitants; whereas, after they had been put in force, scarcely five out of a hundred of those who were removed from the proximity of the infected, were subsequently attacked with plague.*

Muratori informs us that similar measures had been adopted with equal success at Ferrara, in 1630.†

The Board of Health at Constantinople has, for the last eight or nine years, followed out the prophylactic method recommended by Gastaldy and Mead, removing the infected to a hospital, and emptying every house, in which a case occurs, alike of its inhabitants and furniture, having it well cleansed and purified, and not allowing any one to occupy it for the space of a month. It is to the adoption of these means that the board attributes the exemption of Constantinople and the principal ports in Turkey from the plague, since the year 1839. If, in place of acting in this manner, the houses of the infected are condemned with their inmates to a severe quarantine, the result will necessarily be to create fresh foci of pestilential infection, and thus increase the very evil that is vainly sought to be extinguished.

M. Seisson, principal physician to the hospital at Cairo and formerly professor of the School of Medicine at Abouzabel, observes that if, at the time of the appearance of the plague at Cairo, in 1835, after the arrival of the Maltese Giglio, who died of that disease which he had brought from Alexandria, they had dispersed the other members of his family in the country, it would probably have prevented the death of eight or ten persons who, kept by military force within the house, contracted the plague and died. Two persons fled from the focus of infection, of which the quarantine was the cause; they both remained free.

The case of Giglio, adds M. Seisson, does not prove, as it has been said, the contagion of the plague; it only proves the danger of shutting up, in a narrow space, individuals who have been in connection with an infected

---

* Gastaldy, *Tractatus de avertendâ et prostigandâ* (?) *peste.* Bologna, 1684, folio.

† Muratori, *Governo della peste et delle maniere di guardasena.* Modena, 1714, 8vo.

person. It is therefore of the first importance to abolish what are called special quarantines for houses, in which persons have been seized with plague. On the contrary, they should be at once emptied, aired, and purified, to prevent every focus of infection.*

Dr. Mead has quoted from Gassendi a passage wherein this author attributes the frightful mortality of the pestilence that prevailed at Digne in Provence, in 1629, to the severe measures that were taken to prevent the inhabitants from leaving the town and retiring into the neighbouring country.

In 1720 the inhabitants of Marseilles were prohibited, under pain of death, from leaving that city or its suburbs. Hence, doubtless the terrible devastation that ensued.

" And yet," says M. Prus, with an emphatic force of argument that cannot fail to make a deep impression on the public mind, " what would be done in the present day according to existing regulations, if the plague made its appearance in any town of France? It would be isolated by a cordon of troops, for the purpose of preventing any of the inhabitants leaving it; in other words, the unfortunate town would be condemned to retain, and in a concentrated form too, within its bosom all the various causes which serve to develope foci of pestilential infection. Is it then impossible to reconcile the advantages of the public health with the most common laws of humanity? We think not; on the contrary, we are firmly convinced that perfect security may be given to neighbouring towns and to the whole kingdom, by taking the necessary measures to remove the great majority of the inhabitants of the town, in which the plague should appear, from the danger. To obtain results so desirable, it needs only to know how to profit by all that time and experience have taught us of the epidemicity of the disease and of pestilential infection."

*Conclusion.*—" In epidemic foci, the plague is transmissible by the miasms which emanate from the bodies of the infected, and by the foci of infection thereby produced."

Chap. VI.—*Is the plague transmissible beyond, or away from, epidemic foci?*

This, it will be perceived, is the most important of all the questions respecting the history of the plague, in reference at least to the subject of Quarantines; for, upon its solution, the propriety of making any change in existing regulations must depend.

A considerable number of medical men, who have studied the epidemics of 1835 and 1841 in Egypt, will answer it in the decided negative, and for the following reasons:

When the epidemic constitution ceases, all or nearly all the sick recover, and no new attacks occur.

Numerous plague-patients have been, and still are, accumulated in the hospitals and houses; all the conditions favorable to the transmission of the plague by mediate or immediate contact, or to its propagation by miasmatic infection, continue to exist together; and yet, at a period that is almost known and determinate, the epidemic becomes extinguished, and with it the plague ceases entirely.

* M. Seisson. *Lettre addressée au consul general d'Angleterre à Alexandrie en* 1839.

An infected person coming from an epidemic focus is now not more to be feared than a sporadic plague-patient, who, by the consent of all the medical men in Egypt, never occasions any risk.

If it is doubtful whether the clothes and goods of infected persons can transmit the plague in the time of an epidemic, it is certain that, when once this has disappeared, such clothes and goods may be used with impunity.

An epidemic only appears in a country in the train of certain local and atmospheric influences, whose action has been prolonged for a greater or lesser period of time; very commonly too, privations, fatigues, physical or moral troubles have been experienced, in different degrees, by the inhabitants. From these united causes result more or less general predispositions to contract the prevailing disease. Now, when a vessel carries one or more infected persons beyond the focus of infection, she cannot take along with them all the causes, past and present, which are necessary to the development of an epidemic.

It must be confessed that the observations, made in Egypt during the years 1835 and 1841, seem to justify these propositions and the conclusions which flow from them.

Let us now see whether the facts observed on board vessels at sea, and in the Lazarettos of Europe, are in accordance with these conclusions.

Since the year 1720 down to the present period, 25 vessels having the plague on board, have arrived in the ports of France or Italy; 10 at Marseilles, 5 at Venice, 8 at Leghorn, and one at Genoa. We shall confine our remarks to the circumstances connected with the arrivals at Marseilles, the official documentary evidence upon these being much more complete than in the other cases. The years in which these arrivals occurred in 1741, 1760, 1784, 1785, 1786 (*bis*), 1796, 1819, 1825, and 1837. The entire number of cases of plague (omitting all the doubtful ones), treated in the lazaretto of this port since 1720, is 32; and of these, 18 have proved fatal. Three of the quarantine surgeons caught the disease during their attendance on the infected; they all recovered. A fourth surgeon, who had arrived on board an infected ship, and subsequently acted in his professional capacity in the lazaretto, died. Four of the health-guards, who had been (most improperly) put on board infected ships, contracted the disease in the lazaretto; two died. A sailor, who acted as assistant in the lazaretto infirmary, was taken ill and died. Two other sailors, belonging to an infected vessel, but who seemed to have caught the disease in the lazaretto where they had been confined for more than 12 days, died.

In the 11 cases therefore of plague, which might have been contracted in the lazaretto, 6 of the patients recovered, and 5 died: all the latter cases occurred in men who had been on board infected vessels. Of the three health-guards, who had caught the disease on board, only one recovered. Indeed it would seem that, in all the fatal cases, the patients had been for a longer or shorter period of time on board infected vessels.

It appears, also, that not one of the cases, which occurred on board a vessel at sea during the voyage to France, recovered;—a circumstance that very emphatically shews the malignancy of the disease when it occurs in a crowded confined space, and the great advantages of treating it in a large open lazaretto.

From the facts now alluded to, we are surely justified in maintaining not only that the plague may be transmitted on board-ship among individuals coming from the same infected focus, and living in the same hygienic conditions; but also that a plague-patient, received into a lazaretto in another and distant country, may become the cause of infection to others. The *conclusion*, therefore, of the Commissioners is this:

"It is indisputable that the plague is transmissible beyond or away from epidemic foci, whether on board vessels at sea, or in the lazarettos of Europe."

The question proposed in Chap. VII. is to this effect: *Is the plague transmissible, away from epidemic foci, by immediate contact with the infected?* It is at once answered in the negative; there is not a single authentic case on record to prove that the disease has ever been propagated in the way mentioned. The same thing may be said in answer to the question in the following Chapter, viz: *Is the plague transmissible, away from epidemic foci, by the clothes or other effects which have been used by the infected?* Instances indeed have been related, by some writers, of passengers appearing to be taken suddenly ill almost immediately after opening their bags or boxes, and handling their contents; but not one of the narratives of this sort is satisfactory in its details or conclusive in its evidence. Nevertheless the Commissioners, unwilling to commit themselves unqualifiedly on the point under consideration, suggest that new experiments and observations should be made with all possible precautions, at a distance from every focus of infection, and in a locality where the plague is not endemic.

CHAP. IX.—*Can articles of merchandize transport the plague beyond or away from epidemic foci?*

The evidence and facts, on which the advocates (few in number as they are in the present day) of the affirmative side of this question chiefly rest their opinion, are the following:

The plague of London in 1665 is said by Hodges to have been imported from Holland into England in bales of cotton. But it may be fairly objected to this opinion, that the registers of that city clearly shew that the disease had been endemic within its walls for some years preceding the outbreak of the great pestilence, and the destructive conflagration that occurred immediately afterwards. Since that period, the plague has never reappeared in the English metropolis.

The testimony, that has been adduced to shew that the plague of Toulon in 1721 was owing to the reception by some of the inhabitants of some suspected silk that was stolen out of quarantine, is equally unsatisfactory. These and such-like statements cannot be fairly admitted, in the present day, to be entitled to much weight in the determination of the important question under enquiry. We are not sufficiently acquainted with the accompanying circumstances of either case to warrant us in laying much stress upon them. Does the following fact,—the details of which may be relied on as in every respect authentic and perfectly accurate—communicated by Dr. Laidlaw to the English Consul at Cairo, enable us to form any thing like a decided opinion?

In 1835, the epidemic plague raged at Alexandria among all the servants and employés living in the magazines of the Egyptian government.

Notwithstanding this, a vast number of bales of cotton, daily handled by the prisoners, were exported from January to June—that is to say during the whole continuance of the epidemic—to all the great ports of Europe.

There were exported this year

| | |
|---|---|
| To England . . . | 31,709 bales. |
| To Marseilles . . . | 33,812 „ |
| To Leghorn . . . | 424 „ |
| To Holland . . . | 150 „ |
| To Trieste . . . | 32,263 „ |
| To other ports . . . | 32 „ |

Now, although no precautionary means were taken in the way of disinfecting this immense quantity of an article that has always been deemed highly susceptible of retaining the infectious effluvia, not one person seems to have been infected in consequence.

Of sixteen English vessels laden with cotton, which sailed from Alexandria from the beginning of January to the end of June, eight had the plague on board ; and yet their cargoes did not prove more dangerous than those of the non-infected vessels.

Besides this very conclusive evidence, the Commissioners mention upon official authority that, since the year 1720, not one of the porters employed at the lazaretto of Marseilles in discharging and landing the cargoes of suspected ships has ever caught the plague.

The *conclusion* is therefore fairly forced upon us that

"There is nothing to prove that articles of merchandize can transport the disease beyond epidemic foci."

The division of objects of merchandise in the French lazarettos into three classes, according as they are (believed to be) susceptible, doubtfully susceptible, and non-susceptible of infection, is the most arbitrary and ridiculous thing imaginable; nor is it easy even to form a conjecture what possibly could have led any set of reasonable men to adopt it. Tallow and wax, for example, are declared to be non-susceptible objects ; but when made into candles (from the wicks, we suppose), they are susceptible ! Pieces of old copper and other metals are conductors of the pestiferous poison ; wood and other porous substances are not ! Truly, as M. Prus remarks, the classification can only be regarded " as the result of most imperfect observation and of antiquated traditions prompted by fear and prejudice." It is utterly discreditable for any enlightened government to retain and act upon it.

The great point is to determine whether the clothing and baggage of plague-patients are capable of communicating the disease in our ports. If the decision upon this subject be in the affirmative, then certainly it will be right to ascertain what other objects possess the same property; but should it be in the negative, it is scarcely necessary to say that the entire catalogue of interdicted articles must be swept away.

It is quite unnecessary, as a matter of course, to say a single word respecting the comparative value of different modes of disinfection that have been proposed at different times. Most of the substances used in fumigation are utterly worthless ; some of them are dangerous, and therefore inexpedient. Chlorine and its preparations are unquestionably the safest and best.

*Conclusion.*—" The study of the means best fitted to disinfect baggage, clothes, and articles of merchandise, remain still to be made.

" To be rational, before researches on this subject are undertaken, it should be proved that these different objects are really capable of becoming charged with the principle of the plague."

Chap. X.—*Can the plague be transmitted by pestilential miasms, beyond or away from epidemic foci?*

When, on board-ship or in a lazaretto, the plague is communicated from an infected person to one in health, it is obvious that it must be always difficult, and often impossible, to say positively that the disease has been owing to direct contact with the sick; for, whoever has been so near a patient as to have touched his body, must have inspired the pestilential atmosphere that is around him. How, then, shall we decide whether the infection has taken place by the skin or by the lungs? The task is indeed not easy, if indeed it be possible. But if we consider, on the one hand, that numerous observations have clearly shewn that immediate contact with plague patients have not given the disease, and, on the other hand, that residence in a focus of pestilential infection, but without any contact with the sick, has imparted it, we are surely almost compelled to admit the following *conclusions*, viz.

" 1. The transmission of the plague by pestilential miasms is a proved fact.

" 2. The transmission of the disease by immediate contact with the infected is not a proved fact."

It has been already seen, from the experience of the Marseilles lazaretto, that, whenever there was plague existing in a ship, the stay of the vessel in the port became dangerous not only for the crew and passengers, but also for the health-officers who were sent on board, and who, it will readily be believed, most carefully avoided all contact with the sick or with any suspected object. Too often the vessel became a genuine focus of pestilential infection. The air, loaded with the miasms that always emanate from the bodies of the sick, acts as a poison to all who inhale it. This infected state of atmosphere on board a vessel may continue for some time, after every plague-patient has been removed from on board. We are therefore fairly warranted in concluding that " the plague is transmissible by infection (in other words, by the atmosphere being charged with pestilential miasms from the bodies of the sick) beyond or away from epidemic foci, as we have already seen that it is so in epidemic foci, and in countries where the disease is endemic."

To this general conclusion it may be useful to append the following three which are but as corollaries from it, as upon each of them certain quarantine measures are based.

" 1. It follows from the facts adduced in the preceding chapters relative to the transmissibility of the plague, alike within and at a distance from epidemic foci, that plague-patients, by vitiating the air of places wherein they are confined, may create foci of pestilential infection that are capable of transmitting the disease.

" 2. Foci of pestilential infection may persist in a place after the removal of plague-patients from it.

" 3. Foci of pestilential infection once formed in a vessel, by the presence of one or more plague-patients on board, may be transported to great distances."

Chap. XI.—*Is sporadic plague transmissible either by the infected themselves, or by foci of infection which they may form?*

This question has already received a partial answer; but it will be useful to recur to its consideration in this place. The grounds, upon which almost all the medical men in Egypt have adopted a negative opinion on the matter, are as follow.

After the epidemic of 1835 had ceased, the attention of these gentlemen —divided, be it remembered, in opinion as to the general question of the transmissibility of the plague—was naturally directed to the sporadic cases which occurred in the latter half of that year, and also in 1836, 37, and 38. From June 1835 to the end of December 1838, 649 cases of sporadic plague were observed in Alexandria. Now it is admitted by all that, of these 649 cases, 646 did *not* transmit the disease to any of the persons who had waited upon, and had frequently touched, the sick. It is therefore in reference to the three remaining cases only that any difference of opinion has existed; and with respect to these three cases, we think that no unprejudiced reader will hesitate to say, after carefully perusing the reports, that they are very far from proving that the disease was communicated, in a single instance, from one patient to another.

Here it deserves enquiry whether the cases of plague that have at any time been imported into Europe have, or have not, been brought by vessels which had left the producing countries of the disease while a pestilential *epidemic* was prevailing. The imported cases of the plague have never, we believe, been sufficiently considered in this point of view, although it bears very obviously on the question of the transmissibility of *sporadic* plague. The examination of the histories of the ten importations of the plague into Marseilles since 1720 has led the reporter to believe that, in every one of these instances, the pestilence was raging epidemically in the ports whence the vessels had come. This interesting fact certainly does not prove that sporadic plague is not transmissible; it only shows that none of the numerous vessels, which have left Egypt, Syria, or Turkey since the year 1720, at a time when these countries suffered only from sporadic plague, has ever imported the pestilence into Marseilles. This consideration, coupled with the remark of intelligent and trustworthy observers in Egypt that the cases of sporadic plague, which have occurred from July 1835 to the beginning of 1839, have in no instance transmitted the disease, deserves the serious attention of all medical men and legislators. M. Brayer also has come to the same opinion respecting the sporadic plague at Constantinople.

*Conclusion.*—" Patients affected with sporadic plague do not seem to be capable of producing foci of infection sufficiently active to transmit the disease."*

Chap. XII.—*Is the plague more or less readily transmissible according to the intensity of the epidemic, the different periods of its course, and the or-*

* In a subsequent part of the Report however, when treating of quarantine restrictions, it is stated that "the non-transmissibility of sporadic plague is not yet sufficiently determined by experience to warrant us in founding a sanitary measure upon it."

*ganic susceptibilities of the individuals exposed to the action of pestilential miasms ?*

There cannot be a doubt for a moment that different epidemics of this as well as of other pestilences, exhibit very different degrees of intensity or malignancy, as evidenced by the varying amount of mortality produced in different seasons. Now, a question here arises, whether the risk of infection is at all proportionate to the degree of this malignancy. It will be obvious that it is scarcely possible to determine this point within the range or operation of the general exciting causes of the pestilences, or, in other words, of the epidemic focus. The observations, to be at all satisfactory, must be made on cases occurring beyond, or at a distance from, such a focus ; for there, the miasms exhaled from the bodies of the sick will operate alone. The results of past experience seem unquestionably to indicate that the liability of such cases to propagate themselves is in direct relation with the intensity of the epidemic at its place of origin. The greater this intensity, the more readily the disease is communicable ; and the reverse holds true, in proportion as this intensity diminishes. When the plague ceases to be epidemic, its transmissibility appears to cease altogether.

The period, too, of the epidemic has a decided influence on the force of the transmissibility of the disease. We have already considered this point, and need not dwell upon it here.

What has been said respecting the period of the epidemic in this point of view, is equally true of the period of the disease. Larrey was of opinion that the plague was not communicable during its first period or stage. Dr. Lacheze maintains that the disease ceases to be so after the second stage ; that is, after the fourth or fifth day from its invasion. In the case of the disease, as in that of the epidemic, it is the second period which is most dangerous. Moreover, the influence of the organic dispositions or susceptibilities of individuals, in other words of their general state of health and their idiosyncrasies, either in promoting or in counteracting the liability to infection, cannot be disputed by any one. Much unquestionably depends upon the hygienic regime that is followed. Whatever tends to enfeeble or deprave the powers of life, renders the system more liable to infection. Hence excessive fatigues, the want of proper nourishment and the abuse of spirituous liquors on the one hand, and luxurious dissipation and excessive venery on the other, are all powerfully predisposing causes. Larrey observed that any tendency to the scorbutic diathesis rendered the system unusually susceptible, and that all such cases proved very rapidly fatal. We have already seen that race, nationality, sex, age, the circumstance of being acclimated or not, &c. have all some influence in aiding, or otherwise, the tendency to be affected by general epidemic causes. The same is the case with respect to the action of pestilential miasms.

Chap. XIII.—*Is there reason to believe that the plague, when imported from the East into any European port, may be communicated to a sufficiently large number of persons to give rise to a pestilential epidemic ?*

The medical men of Egypt answer this query in the negative. Their opinion is based on the often observed fact that, when plague-patients are transported to places not subject to the pestilential constitution, they die or recover without transmitting their disease to any one. If the infected, as we have seen, cannot communicate the disease to the inhabitants of certain places in Upper Egypt, how shall we believe that, when transported from Egypt to France, it will possess a power of transmission so strong as to occasion an epidemic?

Some observers—and Dr. Lacheze is of this number—have indeed remarked that, in certain cases and in certain localities not subjected to a pestilential constitution, the disease has been communicated to a few individuals; but that these latter in no instance transmitted it to any one, notwithstanding the most free and intimate intercourse. Dr. Lacheze does not admit that the plague can ever, without a pre-existing epidemic influence, attack a sufficiently large number of persons to constitute a public calamity; and he insists that, wherever it has committed great ravages, there has uniformly been a pestilential constitution prevailing at the time. Sydenham, too, was of this opinion; for we find it distinctly asserted in his writings that, however frequent the importation of the plague might be into England, the disease would assume an epidemic character only every thirtieth or fortieth year; because then only would it find the atmospheric conditions and the organic predispositions that are favorable to its development and propagation.

This doctrine is very generally received in Egypt in the present day, and its truth has already been recognised by many of the most intellectual physicians in Europe. The legitimate inference from its public recognition would be that vessels arriving from infected ports, and having the plague on board, might be permitted to land the sick at any place not subject to the epidemic pestilential influence, without any risk to the people of that place, whether the patients recovered or not. But then it must be remembered that, until we are better acquainted than we are yet with what are the conditions of the soil and the atmosphere which, in Europe, may give rise to a pestilential constitution, and what is the meteorological condition in which an imported plague may be liable to become diffused, prudence will authoritatively require that the very same measures should be taken in the ports of France against the possibility of the propagation of the pestilence by the infected, as if there was no room for doubt that they may become the cause and starting-point of a pestilential epidemic.* This caution is more particularly necessary in the case of Marseilles than of any other place in France, in consequence of its many local sources of insalubrity.

This great sea-port presents—in the circumstances of its climate, of its harbour being choked with filth of all sorts and containing an admixture of salt and fresh water, of its large working population, of its being hemmed in on all sides by mountains which prevent the free circulation of the air, and lastly of its proximity to large ponds—the very conditions that are favourable to the development of the plague.† It is a remarkable thing

---

* This is one of the conclusions of M. Prus, the justice of which has very fairly been impugned.—*Rev.*

† *La topographie medicale de Marseille par M. Ducros, medecin en chef de l'Hôtel Dieu de Marseille,* 1837.

that the two cities of Europe which, after Constantinople, have suffered most from this pestilence are Venice and Marseilles :—Venice, which by its lagunes, its filthy canals, the moist heat of its climate, and by the wretched state of a large portion of its inhabitants, combines most of the causes which engender spontaneous plague, and may therefore, *a fortiori*, promote the diffusion of the disease when imported; and Marseilles, which in this respect approaches far too near to Venice.

Here we should not fail to remark that sufficient attention has not hitherto been paid to the cases, certainly very numerous, where the plague conveyed to a country has become spontaneously extinguished, for very want of being able to transmit itself. It would be very useful to ascertain with precision what are the local causes which appear to resist the transmission and propagation of the disease. This work has only just been commenced in respect of a few places in Upper Egypt.

*Conclusion.*—" If it has been proved that the existence of a pestilential constitution in a country, into which the plague is imported, is necessary for the transmission and propagation of the disease, it seems nevertheless certain that imported plague will not exercise any great ravages, if it does not meet with, in the character of the climate, atmosphere and population of the place, the conditions that are favourable for its development."

### Fourth Part.

*What is the ordinary or exceptional term of the incubation of the plague?* —in other words, how long may the plague remain hidden, so to speak, in an infected individual, before it manifests itself by more or less distinct symptoms?

This period is believed to vary considerably according to the stage of the prevailing epidemic, and other less influential circumstances. The variations are nevertheless confined within certain limits, which it is important to ascertain; for upon them should depend, in a great measure, the duration of quarantines.

All observers have remarked that, when a pestilential epidemic commences in a town, the incubation of the disease is often extremely short. We read of attacks of the plague proving fatal within a few hours, nay within a few minutes: these are the cases, which have been truly called " pestes foudroyantes."

In the second period or stage of the epidemic, the usual term of incubation is from three to five days. It is about the same in the third stage.

Upon all these points there is little or no discrepancy of opinion. It is only when we endeavour to determine the longest duration or lapse of time that may be fairly admitted for certain exceptional cases, that we find the statements of different writers to disagree. Some, and these constitute the large majority, maintain that the term of incubation never exceeds eight days; others think that this term may be prolonged to ten days, and sometimes, although very rarely, a few days more. Dr. Grassi, in his reply to certain interrogatories addressed to him in 1839 by the English minister, says upon this subject:

" In the course of several years, some thousands of persons of every age, sex and condition, were condemned to undergo a quarantine of observation of six days for having been exposed to infection. The disease made its appearance in many of them during their isolation, but in no one case beyond the sixth day. This is an observation which I have made with great attention."

On his representations to the Egyptian government, the quarantine, which had before been eleven days in the lazaretto at Alexandria, was reduced in 1842 to seven days.

The experience of other medical men in Egypt has, on the whole, confirmed the truth of M. Grassi's observations.

This gentleman assures us that, among the multitude of people that left Cairo during the epidemic of 1835 and fled into Upper Egypt, which continued healthy, the disease manifested itself in a few persons; but, in no one instance, more than the eight days after their quitting the city.

The observations made, in the course of the same year, by the professors of the medical school at Abouzabel lead to the general conclusion, that the period of incubation never exceeds six days. According to M. Segur, it is never more than eight. If, too, we examine what has occurred in vessels that have had plague on board after leaving an infected port, we shall find that on every occasion the disease has shown itself within eight days from the time of sailing.

With respect to those cases where it has been alleged that the period of incubation exceeded eight or ten days, M. Prus does not consider them at all worthy of acceptance, in the sense in which they have been adduced; for no account has been taken by their narrators either of the epidemic action, or of the miasmatic infection which always acts an important part in places that are badly ventilated; in a ship for example. When a vessel becomes a focus of infection in consequence of having had a number of plague patients on board, the persons, who remain in this focus and breathe the vitiated air, may, and often do, contract the disease at intervals of a longer or shorter space of time. It is obvious that, under such circumstances, the sailors and passengers may be seized 15, 20, 30 days and even upwards, the one from the other, without the inference being at all warranted that the incubation of the malady has existed in any one case for more than six or eight days. We in truth cannot make out when the pestilential miasms began to act on those who had received them into their systems, in such a manner as to occasion the development of the disease.

*Conclusion.*—" If it be true that a fixed and absolute term cannot be assigned to the incubation of the plague, it seems, nevertheless, to be clearly proved by well-established facts that, at a distance from countries where it is endemic and beyond or away from epidemic foci, the disease has never broke out in persons who have been exposed to its influence after an isolation of eight days. The few facts, which might be regarded as exceptional to this rule, are all susceptible of another interpretation."

The concluding part of the Report is occupied with an account of the Quarantine regulations now in force in the different ports of France on vessels arriving from suspected countries, and of the changes which the Commissioners propose should be adopted. We have no intention to allude to the first of these matters; and, with respect to the second, our remarks shall be very brief. It is suggested, and the suggestion is certainly a valuable one, that medical men should be appointed by Government to reside in the different places where the plague is most apt to exist, in order that regular reports as to their sanitary condition might be transmitted home, and for the purpose of officially examining the state of every vessel, with respect to her passengers, crew, cargo, accommodations, &c., about to leave the country for any French port. The resident consular agents would then be better qualified, by the accurate professional details thus acquired, to determine when to grant and when to refuse clean bills of health to the vessels of their own nations. The affixing of the medical certificate to the ship's bill of health would also enable the officers of the port, where the vessel arrived, to judge more correctly of all the circumstances connected with her past and present condition. The Commissioners propose that no clean bill of health should be granted when a pestilential epidemic prevails or appears to be impending in the place of departure, or even when the number of sporadic cases of the plague are so numerous and severe as to occasion apprehensions that the malady may spread. In all other cases, a clean bill might be granted at the port of departure.

Although believing that the clothes and other effects of the sick are not capable of transmitting the plague, the Commissioners suggest that, until further experiments are made upon this subject, all those articles should be duly ventilated during the voyage; or, what would be better, that the trunks and boxes of the passengers and crew should be secured and stamped (plombées) at the port of departure, and not be opened until this can be done in a French lazaretto, where they should be well ventilated for three days or so.

The following are the suggestions of the Commissioners in respect of the Quarantines which they deem advisable, in lieu of those now existing in French ports.

1. For vessels having a medical man on board, and coming from Egypt, Syria, or Turkey with a clean bill of health, the quarantine to be 10 full days, *to date from their departure*, provided no case of plague or of any suspicious disease has appeared during the voyage.

The quarantine to be 15 full days, *to date from their departure*, for the same vessels arriving with a foul bill of health, if neither plague nor any suspicious disease has occurred on board during the voyage.

2. For merchant vessels arriving with a clean bill of health, but having no medical man on board, a quarantine of observation to be for ten full days, *to date from their arrival*.

When the same vessels shall arrive in a port with a foul bill of health, but without having had either plague or any suspicious disease on board while at sea, a most strict quarantine to be for 15 days, *to date from their arrival*.

If a case of plague, or of suspicious disease has occurred on board during the voyage, or should occur after the arrival of the vessel in a French port, the vessel to be subjected to a rigid quarantine, the length of which to be determined by the sanitary authorities of the port. The passengers and crew to be transferred to the lazaretto, and detained for 15 days at least, and 20 at most; the cargo to be unloaded and freely exposed to the air; the vessel herself to be well cleansed out, purified, and left empty for a month at least; and health-guards to be stationed near to, but not to be put on board of, the infected vessel.

With respect to the cargo, since it has not been proved by any authentic fact that articles of merchandize have the property of retaining pestilential miasms, and of transmitting the plague, the Commissioners confine themselves to merely recommending the employment of such means as are most simple and least oppressive or vexatious to commerce.

After exposing the barbarous absurdity of the plan followed at the Marseilles lazaretto, even up to the present day, in the treatment of any one affected with, or supposed to be affected with, plague, it is suggested not only that plague-patients should be treated and waited upon like any other patients, without using the cruel and ridiculous precautions that are still in force, but also that *post-mortem* examinations should be made in all fatal cases.

Should the plague break out in any house, town, or district, the rules to be followed are simple and obvious. The sick should be immediately removed from the dwelling, where the disease has appeared, to a healthy well-aired spot that has been fixed upon ; while all the other inmates also should be compelled to leave it, in order that it may be duly cleansed, purified, and ventilated. On no pretext should the infected be confined and inclosed along with the healthy by sanitary cordons, or other compulsory measures, in the very place where the pestilence exists; this were only aggravating the mischief and rendering it more concentrated. The great object should be to attenuate the virulence of the atmospheric poison by separating the sick from the healthy, and by keeping both in airy, elevated, situations. If necessary, tents and simple barracks should be established, and the healthy compelled to dwell in them, at a distance from the focus of infection.

The Report, of which we have now given so extended an analysis, was elaborately discussed at eight or nine sittings of the Academy of Medicine, in the months of May, June, July and August. Notwithstanding the labour expended, we do not think that either much new matter was added to the details which the Report itself contains, or that any of its more important positions were successfully impugned. The majority of the speakers, as well as of the members of the Commission, declared their cordial concurrence with the results of M. Prus' researches, while the minority were divided into two sections that maintained very opposite opinions; the one advocating almost all the extravagant notions held by the ultra-contagionist party, and the other loudly proclaiming the non-communicability of the plague under almost any circumstances, and calling for the entire abrogation of all quarantines. The reader will be able to judge for himself of the value of the extreme opinions of both parties.

By far the most valid objection to the Report is, that the practical recommendations proposed in its concluding part are not altogether consistent with the doctrines professed before; the quarantines recommended are unnecessarily stringent and severe, if the conclusions in the body of the Report be correct.

From want of space here, we must refer the reader to page 525 for the review of the Parliamentary Correspondence.

